# Nitrate-to-Ammonia Electroconversion at Neutral pH on Polycrystalline Vanadium Sulfide Derived from Vanadium Disulfide

**DOI:** 10.1021/acsaem.5c01047

**Published:** 2025-06-16

**Authors:** Logan M. Wilder, Taylor J. Aubry, Carter S. Gerke, O. Quinn Carvalho, Jonathan R. Thurston, Michael F. Toney, Michelle A. Smeaton, Jao van de Lagemaat, Elisa M. Miller

**Affiliations:** † Materials, Chemical, and Computational Science, 53405 National Renewable Energy Laboratory, 15013 Denver W Pkwy, Golden, Colorado 80401, United States; ‡ Department of Chemistry, Johns Hopkins University, Baltimore, Maryland 21218, United States; § Department of Chemistry, 1877 University of Colorado Boulder, Boulder, Colorado 80309, United States; ∥ Department of Chemical and Biological Engineering, University of Colorado Boulder, Boulder, Colorado 80309, United States; ⊥ Materials Science and Engineering Program, Renewable and Sustainable Energy Institute, Boulder, Colorado 80303, United States

**Keywords:** nitrate reduction, vanadium sulfide, vanadium disulfide, solvothermal growth, ammonia, grand canonical density functional theory, kinetic isotope effect

## Abstract

The electrochemical nitrate reduction reaction (NO_3_RR) offers a pathway to produce NH_3_ for fuel and fertilizer from waste NO_3_^–^. In this work, a polycrystalline vanadium sulfide (VS_*x*_), which is derived from solvothermally grown and annealed VS_2_, is shown to exhibit excellent NO_3_RR activity (2.3 ± 0.6 mg·cm^–2^ geo.·h^–1^ @ −0.92 V_RHE_) and Faradaic efficiency to NH_4_^+^ (69 ± 6% at −0.69 V_RHE_) in buffered neutral pH electrolyte containing 0.1 M NO_3_^–^. A variety of characterization techniques are leveraged to support the VS_*x*_ assignment, including X-ray photoelectron spectroscopy, near-edge X-ray absorption fine structure spectroscopy, selected area electron diffraction, and X-ray diffraction measurements. The VS_2_ annealing step reduces the oxide character and generates VS_*x*_, which, based on the improved NO_3_RR activity, results in the creation of active sites for NO_3_^–^ binding. To help shed light on NO_3_RR on VS_*x*_, VS_2_ is used as a model system, and a grand-canonical density functional theory (GC-DFT) investigation of VS_2_ shows strong evidence that S vacancies are active sites for NO_3_RR, where NO_3_^–^ outcompetes H^+^ for adsorption at the S-vacancy sites. Moreover, GC-DFT results highlight a thermodynamically favorable reaction to generate NH_4_^+^ in an aqueous electrolyte at relevant cathodic potentials. As an annealed material, VS_*x*_ may contain undersaturated V sites, which show an electronic structure similar to the theoretically calculated S-vacancy site of VS_2_, and these sites may contribute to the observed increase in NO_3_RR activity and selectivity for NH_4_^+^ on VS_*x*_ versus unannealed VS_2_. Finally, kinetic isotope effect measurements suggest that the kinetic rate-limiting step of the NO_3_RR on VS_*x*_ is not proton-coupled, indicating it may be the first electron transfer to adsorbed NO_3_*.

## Graphical abstract

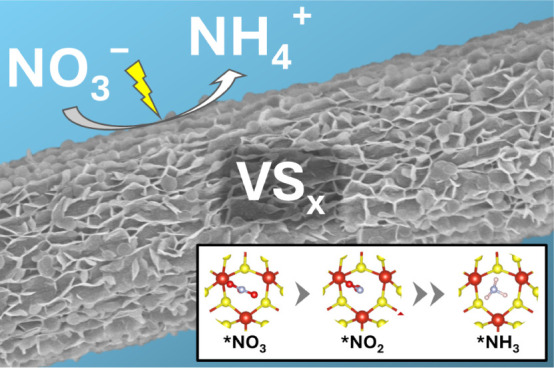

## Introduction

Ammonia (NH_3_) is critical to agriculture and is an energy-dense renewable fuel.^1^ The dominant thermochemical NH_3_ synthesis process (Haber-Bosch) is responsible for ∼1.5% of global CO_2_ emissions.^2^ Additionally, the widespread use of NH_3_-based fertilizer regularly causes destructive NO_3_^–^ pollution in aquatic ecosystems and drinking water sources.^3^ The electrochemical nitrate reduction reaction (NO_3_RR) is a promising solution for NO_3_^–^ remediation, converting NO_3_^–^ waste into fuel or fertilizer and enabling decentralized NH_3_ synthesis under benign conditions while closing the nitrogen cycle. Leveraging NO_3_^–^ as a feedstock (an already oxidized and reactive nitrogen species) eliminates the challenge of overcoming the thermodynamic and kinetic barriers associated with N_2_ activation in the electrochemical nitrogen reduction reaction, offering a more accessible pathway to NH_3_ synthesis. The NO_3_RR could also facilitate NH_3_ synthesis from N_2_ if an energy-efficient process for electrochemical N_2_ oxidation is developed.^4^ Despite progress, study of the NO_3_RR on early-series transition metal catalysts remains underexplored, with only a limited number of catalytic materials reported to date, underdeveloped optimization strategies across catalyst systems, and limited understanding of active site composition.^5^ Thus, advancing the NO_3_RR requires the development of efficient, stable catalysts alongside deeper mechanistic insights and material design principles.

In recent years, transition metal-based catalysts have achieved some of the highest Faradaic efficiencies for the NO_3_RR.^6–9^ However, achieving high current density at low overpotential (η < 0.4 V) remains a significant challenge.^10^ Vanadium-based electrocatalysts have shown promising NO_3_RR selectivity and activity.^11–14^ Additionally, vanadium plays a role in biocatalysis for NO_3_^–^ reduction, as evidenced by the existence of vanadium-based NO_3_^–^ reductase enzymes,^15^ presenting an opportunity for biomimetic electrocatalysis. Vanadium offers a wide range of oxidation states in many amorphous and crystalline sulfides, enabling high tunability of vanadium-sulfide-based electrocatalyst electronic structure.^16^ In a report by Wang and coworkers,^14^ lanthanum doping was employed to enhance the activity of the transition metal dichalcogenide (TMDC) VS_2_ toward NO_3_RR. TMDCs such as VS_2_ have gained widespread interest as catalytic materials due to the high surface area microstructures derived from their layered nanostructure and their tunability through the introduction of vacancies, dopants, and/or adatoms.^17,18^ Other vanadium sulfide stoichiometries have been less explored for the NO_3_RR and remain an area of opportunity. A table listing the performance of several vanadium sulfide-containing catalysts for the NO_3_RR is provided in Table S1.

In this report, a vanadium sulfide electrocatalyst derived from the layered VS_2_ is presented. Polycrystalline vanadium sulfide (VS_*x*_) is generated from a solvothermally grown polycrystalline vanadium disulfide (VS_2_) by annealing in Ar. The VS_*x*_ and VS_2_ materials are investigated using electronic structure-sensitive methods, including near-edge X-ray absorption fine structure (NEXAFS) spectroscopy and X-ray photoelectron spectroscopy (XPS), as well as structure-sensitive methods, including transmission electron microscopy, selected area electron diffraction (TEM-SAED), and X-ray diffraction (XRD) measurements. Both materials are tested for NO_3_RR by linear sweep voltammetry (LSV) and bulk electrolysis experiments. The mechanism of NO_3_RR on VS_*x*_ is investigated by density functional theory (DFT) simulation of NO_3_^–^ binding and reduction by multiple pathways on the model material VS_2_ with an S vacancy, as well as experimentally by kinetic isotope effect (KIE) measurements.

## Experimental Section

### Chemicals and Materials

Ammonium vanadate (NH_4_VO_3_), thioacetamide, vanadium­(III) oxide, sodium hypochlorite solution (10–15%), potassium phosphate monobasic, potassium phosphate dibasic, sodium citrate tribasic, salicylic acid, deuterium oxide (99.9 atom % D), and sodium nitroferricyanide­(III) dihydrate were purchased from Millipore Sigma (Burlington, Massachusetts). 4-Aminobenzenesulfonic acid and N1-(naphthalen-1-yl)­ethane-1,2-diamine dihydrochloride were purchased from Oakwood Chemical (Estill, SC). Vanadium­(V) oxide was purchased from US Research Nanomaterials (Houston, TX). Ethanol, octylamine, and nitric acid were purchased from Thermo Fisher Scientific (Waltham, MA). AvCarb MGL 370 carbon paper and PiperION anion exchange membrane (80 μm, self-supporting) were purchased from Fuel Cell Store (Bryan, TX). All aqueous solutions and electrolytes were prepared using deionized (DI) water (18.2 MΩ·cm, Milli-Q Gradient System, Millipore Sigma). All materials were used as received without further purification.

### Synthesis of VS_2_ and VS_*x*_

To synthesize VS_2_, ammonium vanadate (0.526 g) and thioacetamide (1.687 g) were added to octylamine (40 mL) in a Teflon Parr reactor sleeve inside an N_2_-purged glovebox.^19^ The resulting suspension was stirred for 1 h. Next, carbon paper (6 cm × 3 cm, AvCarb MGL370) was placed in the solution. Approximately one-third of the carbon paper resided in the solution, and the carbon paper was held vertically. The Parr reactor was closed, and the assembly was removed from the N_2_ glovebox and heated to 160 °C for 18 h. After the heating step, the reactor was cooled to room temperature for over 2 h. The coated carbon paper was removed from the reactor and rinsed with ethanol (10 s), DI water (10 s), and then ethanol again (10 s) to afford VS_2_. Several methods have been used to generate sulfur vacancy sites (S_v_) in VS_2_ including thermal methods such as Ar and H_2_ annealing, as well as chemical methods during synthesis.^20^ Previous reports suggest that S_v_ may be generated in VS_2_ by annealing at high temperature under inert atmosphere,^21,22^ and so, in an attempt to generate S_v_ on VS_2_, the material was annealed under Ar atmosphere at 500 °C for 2 h to afford VS_*x*_.

### Electrochemical Experiments

To evaluate VS_2_ and VS_*x*_ as NO_3_RR electrocatalysts, electrochemical performance was assessed using LSV, bulk electrolysis experiments, and KIE measurements. Electrochemical experiments were conducted in H-cell architecture electrochemical cells (16.0 mL catholyte volume) with a PiperION A-80 anion exchange membrane separator.^23^ The electrolyte for experiments is 0.1 M phosphate buffer (pH 7.0) with added 0.1 M KNO_3_, unless otherwise specified. The multichannel potentiostat used is a BioLogic SP-300 (BioLogic, Seyssinet-Pariset, France). The LSV was performed with a scan rate of 20.0 mV·s^–1^. Immediately prior to LSV, electrodes were subjected to cyclic voltammetry (3 cycles) between –1.0 and 0.2 V_RHE_ with a scan rate of 20.0 mV·s^–1^. For bulk electrolysis experiments using VS_2_, a cleaning step was used prior to the bulk electrolysis experiment to remove solution-accessible intercalated small molecules such as NH_3_. In the cleaning step, VS_2_ was immersed in the same electrolyte as used in the subsequent bulk electrolysis experiment, and chronoamperometry at the same potential as that used in the bulk electrolysis experiment was applied to the VS_2_ electrode for 200 s. Then, the VS_2_ was rinsed with DI water, and finally, the bulk electrolysis experiment was immediately started. In bulk electrolysis experiments and KIE experiments, forced convection was incorporated using a Teflon-coated stir bar (2 mm × 5 mm) with a stir rate of 1000 rpm for bulk electrolysis experiments and 1300 rpm for kinetic isotope effect experiments. All electrochemical measurements were performed with active IR compensation (85%), and reported *E* values are after-the-fact corrected for the remaining 15% uncompensated resistance.

### Quantification of NO_3_RR Products

To quantify NH_4_^+^, an indophenol test was used. The exact procedure used is described in our previous publication.^24^ To quantify NO_2_^–^, the Griess assay was used.^25^ Briefly, a stock solution was prepared by adding 0.25 g of 4-aminobenzenesulfonic acid, 2.5 mg of N1-(naphthalen-1-yl)­ethane-1,2-diamine dihydrochloride, and 2.5 mL of glacial acetic acid to 50 mL of water. Then, 2.00 mL of the stock solution and 0.50 mL of the analyte solution were mixed, and after 10 min, the absorbance at wavelength 545 nm was measured to assess NO_2_^–^ concentration.

### Instrumentation for Characterization of Materials

Scanning electron microscopy (SEM) characterization used a Hitachi S-4800 Scanning Electron Microscope operated at 3.0 kV. For transmission electron microscopy (TEM) characterization, VS_2_ and VS_*x*_ were deposited on TEM grids by mechanical transfer. TEM imaging and selected area electron diffraction (SAED) data were collected using a Thermo Fisher Scientific (TFS) Tecnai F20 S/TEM and a TFS Spectra 200 S/TEM, both operated at 200 kV. Scanning transmission electron microscopy energy-dispersive spectroscopy (STEM-EDS) data were acquired using a TFS Super-X detector. X-ray diffraction patterns were collected with a Bruker D8 Discover equipped with a 2-dimensional area detector using Cu Kα radiation. X-ray photoelectron spectroscopy (XPS) data were obtained on a PHI VersaProbe III instrument using Al Kα radiation (1486.7 eV). The XPS data were calibrated with Au or Cu metal, which was cleaned via Ar-ion sputtering. The XPS-measured raw atomic concentration has a 5% error due to surface inhomogeneities, surface roughness, and literature sensitivity values for peak integration.

NEXAFS measurements were taken at the Stanford Synchrotron Radiation Lightsource beamline 8–2 using total electron yield (TEY) by measuring the drain current and total fluorescence yield (TFY) collection modes at the V L-edges and O K-edge. Samples were measured under UHV (<10^–8^ Torr) in duplicate at two positions at an incident angle of 55° to eliminate orientational effects. The signals were collected simultaneously with the incident beam intensity (i0) using a freshly coated gold grid directly upstream of the sample. TEY and TFY spectra were normalized by i0 to eliminate incident beam fluctuations and optics absorption. The duplicate spectra were confirmed to be the same and were then averaged. The pre-edge region before the V L_3_-edge was subtracted with a linear baseline, and then the L_2_-postedge region was normalized to 1. The L_3_-edge of the NEXAFS spectra was fit with a series of Gaussians using the LMFIT package^26^ in a custom Python script. A peak at ca. 520 eV was also used to account for the contributions from the overlapping L_2_-edge.

### Calculation Details

All reported density functional theory calculations were performed in the grand canonical ensemble using the open-source JDFTx software package (version 1.7.0).^27^ We employ the Strongly Constrained and Appropriately Normed (SCAN)^28^ semilocal exchange-correlation functional, built-in norm-conserving SG15 pseudopotentials,^29^ and a 45 Ha planewave cutoff with spin-polarization effects included. Solvation effects were modeled using the minimally empirical CANDLE solvation model,^30^ with an electrolyte concentration of 0.1 M, consistent with our experimental conditions. Potentials were referenced to the calibrated absolute offset of 4.66 eV for the CANDLE model, and these standard hydrogen electrode (SHE) potentials were converted to the reported reversible hydrogen electrode (RHE) potentials by using pH 7, again consistent with our experimental conditions.

The primitive cell of VS_2_ was first fully optimized using a 12 × 12 × 1 k-point grid. To create a model of the surface, a 4 × 4 × 1 supercell was created and sampled with a 3 × 3 × 1 k-point . Vacancy structures were generated by removing sulfur from the surface. To create a model of the edge, a 3 × 6 × 1 supercell was created and sampled with a 4 × 2 × 1 k-point grid. To reduce periodic interactions, images were separated by at least 10 Å in aperiodic directions, with Coulomb truncation applied in those directions as well as JDFTx’s Coulomb truncation embedding, which computes the Coulomb interaction in a double-sized box. These supercells were then allowed to relax.

Force and energy optimizations were converged to within 1 × 10^–4^ Ha/bohr and 1 × 10^–5^ Ha, respectively. Initial supercell cuts and adsorption structures were generated using Pymatgen.^31^ After full relaxation of the edge and surface structures, only adsorbate and VS_2_ atoms within a 3.5 Å radius of the adsorption site were allowed to relax. This approach was used to eliminate unphysical periodic distortions in the VS_2_ layer, ensuring a more accurate representation of the local atomic environment.

Bond orders were computed using the density-derived electrostatic and chemical (DDEC6) method using the Chargemol program (version 09-26-2017),^32^ which involves spherical averaging of the atomic electron density obtained from the DFT calculations. Previous work has shown that the DDEC6-derived bond orders exhibit good reliability across a broad range of materials.^33^

## Results and Discussion

### Characterization of Materials

Characterization described in the following paragraphs suggests that heating solvothermally grown VS_2_ under an inert atmosphere to 500 °C generates a material that is dissimilar in structure from the synthesized VS_2_, which we label as VS_*x*_. Specifically, the VS_*x*_ shows a reduced oxide content and a different V and S chemical environment in comparison to VS_2_. Scanning electron microscopy images of both materials are shown in Figure 1. Comparison of VS_2_ and VS_*x*_ shows little difference in morphology (Figure 1a,b,e,f) and shows distinct morphology from that of the bare carbon paper substrate (Figure S1). The corrugated catalyst layer is ∼500 nm thick, as determined by a cross-sectional SEM image (Figure S2).

**Figure 1:**
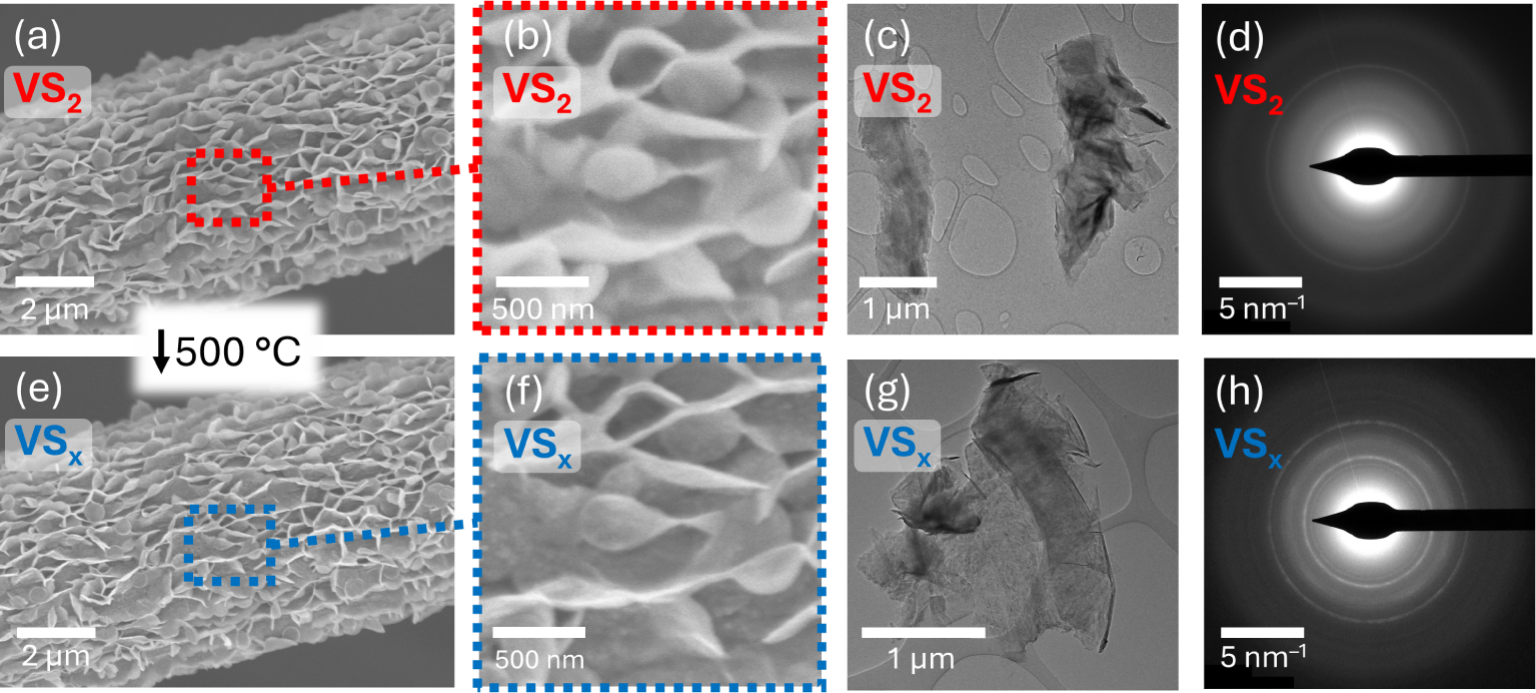
(a,b) SEM images of VS_2_ and (e,f) VS_*x*_, and, (c,d) TEM image and SAED pattern of VS_2_ and (g,h) of VS_*x*_. The VS_2_ and VS_*x*_ SEM images were collected by analyzing the same sample in the same location before and after the annealing step.

Characterization of VS_2_ by TEM and SAED (Figure 1c,d) shows a highly polycrystalline character, and no single-crystal material is observed in any surveyed region. In comparison, the SAED of VS_*x*_ (Figure 1h) reveals a higher crystalline quality, as evidenced by the sharpening of diffraction spots/rings. The VS_*x*_ sample remains largely polycrystalline, though TEM imaging shows some single-crystal flakes (Figure S3) on the scale of 10s of nm. Lattice spacings are measured for both VS_2_ and VS_*x*_ by SAED, and matching lattice spacings of VS_*x*_ are additionally measured from the fast Fourier transform of the TEM image in Figure S3. The SAED patterns were recorded for multiple sample areas, and the measured lattice spacings from SAED measurements are provided in Table S2 and Figure S4, along with an associated discussion. Unambiguous indexing of the SAED-measured lattice spacings of VS_*x*_ and VS_2_ to a single vanadium sulfide phase is not possible due to overlap with multiple reported vanadium sulfide structures, as illustrated in Figure S4. Also, scanning transmission electron microscopy energy-dispersive spectroscopy (STEM-EDS) mapping of VS_*x*_ is provided in Figure S5, which shows that V and S are collocated within the material. The STEM-EDS-reported V:S ratios, as well as the SEM-EDS V:S ratios of VS_2_ and VS_*x*_, are shown in Table S3.

Additional structural information is collected through XRD measurements (Figure S6). The XRD pattern of VS_*x*_ shows only a single intense peak, which could be indexed to the (011) lattice plane of VS_2_ (ICSD 86519), the (111) lattice plane of V_3_S_4_ (ICSD 602568), or the (111) lattice plane of V_5_S_4_ (ICSD 73001). The XRD characterization of VS_2_ indicates that this material shows interlayer-expanded VS_2_-like character. Further discussion of the XRD data is provided in the Supporting Information.

The VS_2_ and VS_*x*_ V oxidation character and bonding environment are investigated by NEXAFS measurements at the V L- and O K-edges using surface-sensitive (∼5 nm) total electron yield (TEY, Figure 2a) and bulk-sensitive (>100 nm) total fluorescence yield (TFY, Figure S7) detection modes. The NEXAFS spectra in Figure 2a contain 3 regions of interest: the V L_3_-edge from 512 to 520 eV, the V L_2_-edge from 520 to 530 eV, and the O K-edge above 530 eV. Comparison of the V L_3_-edge peak maxima of commercially available V^3+^ and V^5+^ oxides versus a V^4+^ sulfide (single-crystal VS_2_, previously reported^34^ and reproduced with permission) shows a predictable distribution, with the oxide peaks occurring at higher energy. This is a result of oxides displaying stronger ligand field strength versus sulfides, resulting in a larger energy gap between the V d-orbitals in V oxides compared to V sulfides.^35^ Our VS_2_ NEXAFS shows features similar to both the sulfide and the oxide reference samples through fitting (Figure S8 and Table S4), including a peak at ca. 516.5 eV and a prominent shoulder peak at ca. 518 eV. Annealing to 500 °C (VS_*x*_) results in a loss of peak intensity at ca. 518 eV and a shift of the ca. 516 eV peak to lower energy. The reduction in the 518 eV peak, along with the decreased intensity of the O K-edge peaks, is consistent with a loss of oxide upon annealing VS_2_ to derive VS_*x*_. The shift of the 516 eV peak to a lower energy upon annealing suggests a slightly different V–S environment. The peak shapes in the TFY spectra (Figure S7) of VS_2_ and VS_*x*_ are similar to the TEY spectra, indicating that the V character in the bulk and surface environments is similar.

**Figure 2:**
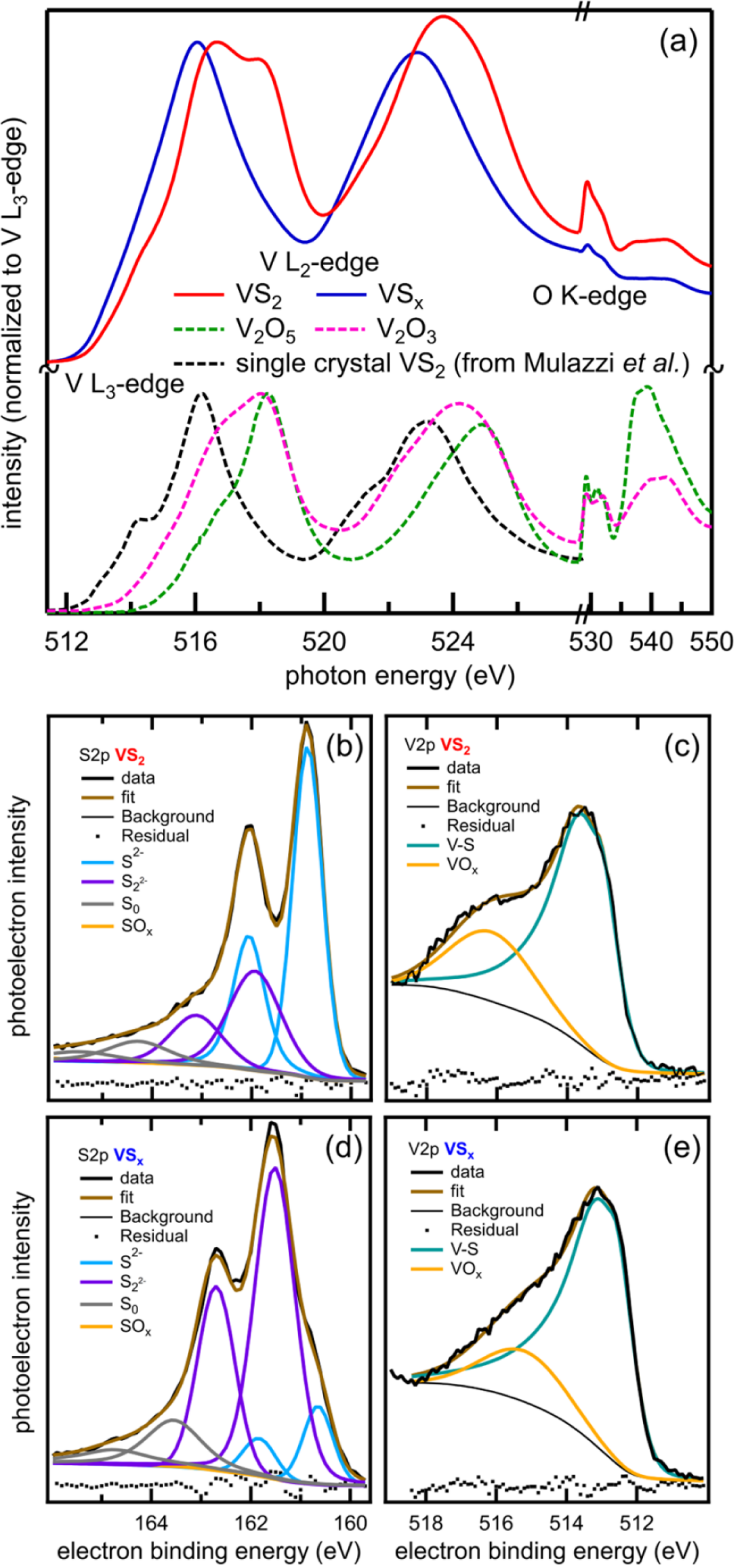
(a) NEXAFS TEY V L- and O K-edge spectra of VS_2_ (red), VS_*x*_ (blue), V_2_O_5_ (green, dashed line), V_2_O_3_ (pink, dashed line), and single crystal VS_2_ (black, dashed line, reproduced with permission from Mulazzi et al.,^34^ and (b–e) XPS spectra of VS_2_ (b,c) and VS_*x*_ (d,e) showing the V 2p_3/2_ and S 2p transitions.

The XPS data are consistent with the NEXAFS analysis and offer additional insight about the S environment and surface stoichiometry (∼10 nm). The XPS data for VS_2_ and VS_*x*_ contain the spin-orbit transition for V 2p_3/2_ (Figure 2c,e). Figure S9, showing an expanded energy range that also contains the V 2p_1/2_ peak, is provided in the Supporting Information. For analysis, we use the more intense V 2p_3/2_ peak to understand the different V environments and oxidation states. In both samples, the peak in the range of 516–517 eV is due to VO_*x*_ (reference V_2_O_3_ and V_2_O_5_ data are shown in Figure S9).^34,36,37^ The trend in the VO_*x*_ is similar to that of the NEXAFS, in that annealing reduces the amount of VO_*x*_. The lower energy peaks in the range of 513–514 eV are due to V–S environments. Similar to the NEXAFS V data, the VS_*x*_ sample shifts to lower binding energy compared to the VS_2_ sample (peak positions and shifts are provided in Table S5). To better understand the change in the V–S environment, we next considered the S 2p core level (Figure 2b,d).

The S 2p core level also contains both spin-orbit peaks, S 2p_3/2_ and 2p_1/2_. A noticeable difference between the two samples is that the VS_2_ sample is dominated by a lower binding energy S environment, and the VS_*x*_ sample has a different peak distribution (fittings in Figure 2b,d as well as peak positions and ratios in Table S5). For the VS_2_ sample, S 2p spin-orbit peaks at 161.0/162.3 eV are the most intense, and we attribute these to VS_2_ (labeled as S^2–^).^37^ Upon annealing, we observe a growth of a higher binding energy S environment (161.8/163.0 eV) for the VS_*x*_ sample. This change in the S peak distribution, along with the V shift with annealing, suggests that the VS_2_ (partially) converts into VS_*x*_, which is consistent with assignments for MoS_*x*_ S 2p, which exhibits a combination of S^2–^ and S_2_^2–^ oxidation states.^38–40^ The S^2–^ state can be comprised of both VS_2_ and VS_*x*_ character.

From the XPS data, we can deconvolute the V peaks into oxide and sulfide components that give the S/V ratio, including either V from the combined oxide and sulfide or the sulfide only. The stoichiometry of VS_2_ and VS_*x*_ as assessed by XPS, along with related discussion, is provided in Table S5. We note that the peak assignments for vanadium sulfide and, in particular, VS_2_ in the literature are not consistent and vary drastically. Therefore, we use our XPS experience with MoS_2_,^41^ literature XPS of MoS_2_/MoS_*x*_,^39,40^ literature XPS of single-crystal-grown VS_2_,^34^ reference XPS of V_2_O_3_ and V_2_O_5_ commercially purchased samples (Figure S9), XPS data fitting, and other characterization methods to gain confidence in these peak assignments.

The XPS data agree with the characterization methods previously discussed, and all of the characterization data suggest that the solvothermally synthesized VS_2_ is dominated by VS_2_-like character, but upon annealing, other vanadium sulfide phases (VS_*x*_) dominate. The annealing step alters the stoichiometry, binding environment, and oxidation states of V and S, which is an unexpected conclusion. In addition to these changes, the generation of S_v_ in VS_*x*_ at 500 °C in an inert atmosphere is possible and supported by previous literature reports;^21,22^ however, vacancies could not be experimentally confirmed in this study.

### Electrochemical Characterization of VS_2_ and VS_*x*_ Catalyst Performance

Electrochemical measurements are conducted in phosphate-buffered neutral pH electrolytes, being the most relevant pH to naturally abundant NO_3_^–^ rich wastewater.^42^ The presence of phosphate in the electrolyte further parallels the conditions of natural waters NO_3_^–^ remediation, as phosphates often occur concurrently with NO_3_^–^ in fertilizer runoff.^43^ In the absence of NO_3_^–^, LSV characterization of VS_2_ and VS_*x*_ in phosphate buffer (pH 7.0) shows moderate-to-poor HER activity for both catalysts (Figure 3a), with the onset of cathodic current (measured at −0.5 mA·cm^–2^ geometric surface area (geo.)) occurring for VS_2_ and VS_*x*_ at −0.63 and −0.57 V_RHE_, respectively. Both catalysts achieve mass-transfer limitations in phosphate by ca. −0.7 V_RHE_,^44^ as evidenced by increased current with the incorporation of convective mass transfer (stirring, Figure S10). In the presence of NO_3_^–^, VS_2_ and VS_*x*_ both show an onset of cathodic current at increased potential, but the difference is greater in VS_*x*_ (Δ170 mV) versus VS_2_ (Δ90 mV). The cathodic wave shoulder resulting from phosphate-coupled proton transfer appears to be retained in both cases but occurs over a broader range. LSV characterization of the carbon paper substrate alone showed some NO_3_RR activity. A version of Figure 3a,c, which reports current density calculated with electrochemically active surface area (ECSA) instead of geometric surface area, is provided in the Figure S11, and characterization of ECSA is shown in Figure S12.

**Figure 3:**
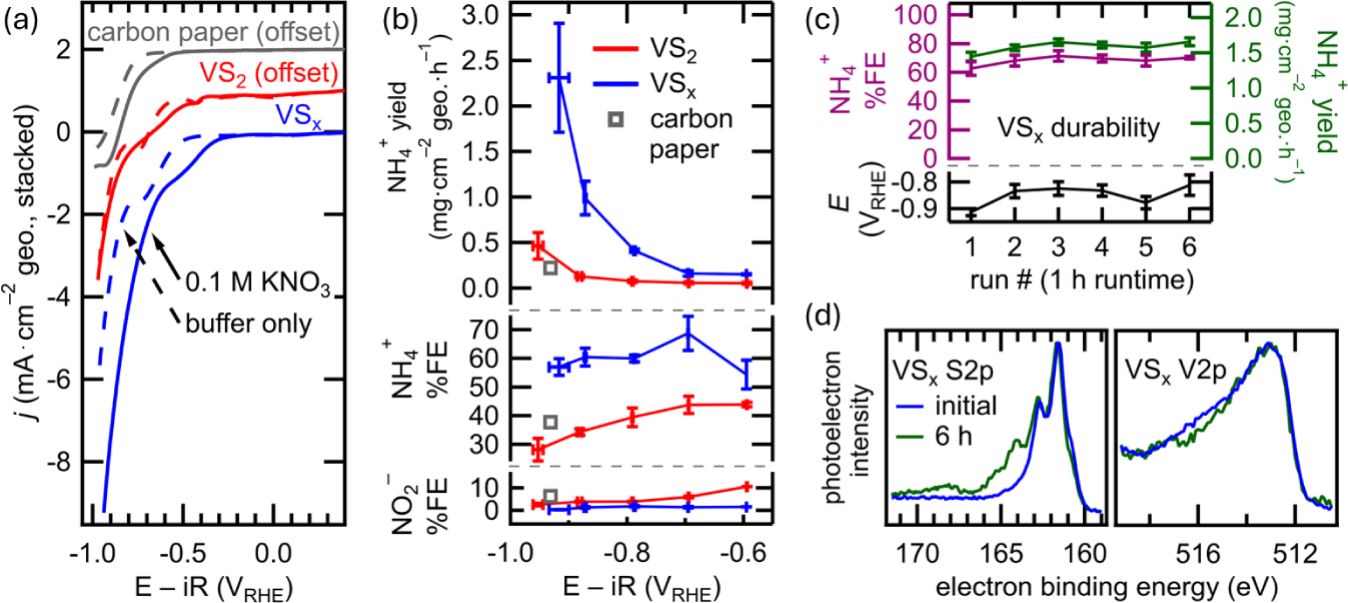
(a) LSVs of VS_2_ (red, offset by +1 mA·cm^–2^ geo.), VS_*x*_ (blue), and the carbon paper substrate alone (gray, offset by +2 mA·cm^–2^ geo.) in Ar-purged potassium phosphate buffer (pH 7.0, dashed lines) and in potassium phosphate buffer with added KNO_3_ (0.1 M, solid lines), measured at 20 mV s^–1^. (b) NH_4_^+^ yield as a function of applied potential (top traces), NO_3_RR Faradaic efficiency to NH_4_^+^ (middle traces) and NO_2_^–^ (bottom traces), (c) NH_4_^+^ yield and Faradaic efficiency to NH_4_^+^ during a 6 h durability test of VS_*x*_ at constant current of 30 mA·cm^–2^geo. in potassium phosphate buffer (pH 7.0) with added 0.1 M KNO_3_, and (d) XPS characterization of VS_*x*_ before (blue trace) and after (green trace) 6 h durability test shown in (c). In (b) and (c), error bars represent the variation (standard deviation) of three replicate bulk electrolysis experiments carried out on the same day.

To further understand the selectivity and activity of the VS_*x*_ and VS_2_ catalysts, bulk electrolysis experiments are carried out by chronoamperometry in stirred electrolyte in a H-cell architecture. The results of the selectivity and activity testing are shown in Figure 3b, and the chronoamperometry traces are shown in Figure S13. A clear difference in selectivity toward NH_4_^+^ generation between VS_2_ and VS_*x*_ is evident in Figure 3b. The Faradaic efficiency (FE) for NH_4_^+^ generation by VS_2_ is <45% at all tested potentials and is <30% at the most cathodic potential. In contrast, VS_*x*_ demonstrates FE to NH_4_^+^ >55% across all tested potentials, reaching a maximum of 69 ± 6% at −0.69 V_RHE_. The maximum measured activity of VS_*x*_ is 2.3 ± 0.6 mg·cm^–2^ geo.·h^–1^ (−0.92 V_RHE_), which is several times higher than the maximum measured activity of VS_2_. In all tested conditions for VS_*x*_, FE to NO_2_^–^ is <2%, however, it should be noted that NO_2_^–^ desorption and subsequent adsorption during the 1 h experimental run cannot be ruled out. The calculated remaining NO_3_^–^ at the end of the 1-h bulk electrolysis experiments is >80% in all scenarios (Figure S14), assuming that the balance of current not contributing to NH_4_^+^ or NO_2_^–^ generation results in the hydrogen evolution reaction (HER). Characterization of the post-bulk electrolysis test VS_2_ and VS_*x*_ catalysts by SEM reveals some loss of VS_2_ catalyst material due to flaking (Figure S15). Bubble formation is visibly apparent during the bulk electrolysis experiments, with increased bubble formation observed at the lowest tested potentials, likely due to the HER side reaction. This bubble formation could contribute to the observed flaking.

Testing VS_*x*_ with decreased NO_3_^–^ ion concentration relative to the results described above (by an order of magnitude, 0.01 M, nominal *E* = −0.9 V_RHE_, Figure S16) decreases activity by roughly an order of magnitude (from 1.0 ± 0.2 to 0.14 ± 0.01 mg·cm^–2^ geo.·h^–1^) and decreases FE to NH_4_^+^ (from 60 ± 3% to 28 ± 4%). Increasing the NO_3_^–^ ion concentration from 0.1 to 1.0 M and testing at the same nominal potential (*E* = −0.9 V_RHE_) shows a different trend, with activity only tripling (to 3 ± 1 mg·cm^–2^ geo.·h^–1^) and FE to NH_4_^+^ showing no apparent difference (59 ± 4%). This concentration dependence suggests that above 0.1 M NO_3_^–^ ion concentration, the NO_3_RR may begin to be limited by available NO_3_^–^ ion binding sites on VS_*x*_, meriting further investigation. A similar concentration-dependent behavior was observed by de Groot et al.^45^ when determining the rate order of NO_3_RR with respect to NO_3_^–^ ion concentration on Pt. Testing VS_*x*_ under acidic conditions (0.1 M HNO_3_) at –0.95 V_RHE_ shows a yield lower than in neutral pH electrolyte but still significant, 1.1 mg·cm^–2^ geo.·h^–1^, and 55% FE to NH_4_^+^ is achieved, suggesting that VS_*x*_ could perform well as a NO_3_RR catalyst in a wide pH range from acidic to neutral pH.

To assess the durability of the VS_*x*_ catalyst, a constant current (30 mA·cm^–2^geo.), 6 h run is carried out, and the results of the durability test are shown in Figure 3c. The experimental conditions for the durability test are identical to the bulk electrolysis testing shown in Figure 3b, with phosphate-buffered electrolyte (pH 7.0) containing 0.1 M NO_3_^–^ used, and the electrolyte is replaced each hour. As shown in the figure, the yield and Faradaic efficiency for NH_4_^+^ do not decrease over the course of the 6-h trial and the potential also does not significantly decrease. Post-durability XPS characterization, shown in Figure 3d, reveals that the V atomic environment remains nearly identical, while the S atomic environment changes slightly over the 6-h run, with additional S peaks observed at ∼164 and 168 eV. The spectral features at ∼164 eV could be S_0_, and the peak at 168 eV is likely SO_*x*_; however, these changes do not drastically alter the V environment. Characterization of the postdurability test VS_*x*_ by SEM reveals some loss of catalyst material due to flaking (Figure S15). Similarly to the 1-h bulk electrolysis experiments, some bubble formation is visibly apparent during the durability test, likely due to the HER side reaction. This bubble formation could lead to the observed flaking.

Control bulk electrolysis measurements of VS_2_ and VS_*x*_ in the absence of NO_3_^–^ show negligible NH_4_^+^ accumulation (Table S6). Tests of the carbon paper support at neutral pH showed a moderate-to-low catalytic activity (Figure 3b,c), which is unsurprising, as NO_3_RR activity has previously been observed on carbon paper electrodes.^46^ At the most cathodic potentials tested, the activity and FE toward NH_4_^+^ are much greater for VS_*x*_ than for the carbon paper, suggesting that the kinetics of VS_*x*_ dominate. In contrast, the carbon paper FE toward NH_4_^+^ is comparable to VS_2_, suggesting that the observed NO_3_RR on VS_2_ could be convoluted by the kinetics of the NO_3_RR on exposed carbon paper.

### Theoretical Calculations of Stoichiometric VS_2_ and Sub-Stoichiometric VS_2_ for NO_3_^–^ Binding and NO_3_RR Activity

To gain understanding of potential active sites for NO_3_RR on VS_*x*_ and the thermodynamics of NO_3_^–^ production pathways, we modeled the metallic 1T-phase of VS_2_ using Grand-Canonical Density Functional Theory (GC-DFT).^27,47^ In this work, 2D slab models are used to represent the surface of the layered VS_2_ material. While this represents an idealized model, this approach is widely used in computational studies of van der Waals materials, in which surface reactivity can be effectively captured using periodic slabs that expose the basal plane or edge terminations of interest. Prior studies have successfully used such models to capture the surface chemistry of VS_2_ and provide a representation of local active sites.^48,49^

T-phase VS_2_ is a correlated material,^50^ and so we employed the Strongly Constrained and Appropriately Normed (SCAN) exchange-correlation functional,^28^ a meta-generalized gradient approximation (GGA) functional incorporating kinetic energy density to better capture weak interactions. SCAN accurately simulates complex materials like strongly correlated cuprates^51,52^ as well as transition-metal oxides,^53^ and it has been validated for 2D materials.^54^ To further describe the electrified interfaces relevant to electrocatalysis, we utilized GC-DFT in combination with the Charge-Asymmetric Nonlocally Determined Local-Electron (CANDLE) solvation model.^30^ Unlike conventional DFT, GC-DFT allows the electron count to fluctuate in response to an applied chemical potential, thereby capturing potential-dependent effects. This self-consistent approach optimizes the grand free energy (Φ) at a given potential, where Φ = *A* – μ*N* (A being the Helmholtz free energy, μ the chemical potential, and *N* the electron count), and provides a rigorous yet computationally efficient framework for modeling solvated electrochemical systems. GC-DFT has provided critical insights for a variety of electrochemical reactions such as nitrogen reduction,^55–57^ CO_2_ reduction,^58,59^ and oxygen evolution.^60^ Further calculation details are provided in the Supporting Information.

To assess active site candidates, we calculated the binding energies of NO_3_^–^ across several VS_2_ models. GC-DFT calculations demonstrate that NO_3_^–^ prefers to adsorb onto under-coordinated VS_2_ basal and edge sites but fails to adsorb on fully S-coordinated VS_2_ basal planes (Figure S17). We therefore consider the thermodynamics of NO_3_^–^ adsorption and reduction to NH_4_^+^ on under-coordinated VS_2_ basal planes. Figure 4a shows the converged structures of our model of the S_v_ sites, corresponding to VS_1.94_, with monodentate and bidentate NO_3_^–^ adsorbed. The monodentate configuration is energetically preferred by ∼1 eV over the bidentate configuration across all potentials considered.

**Figure 4:**
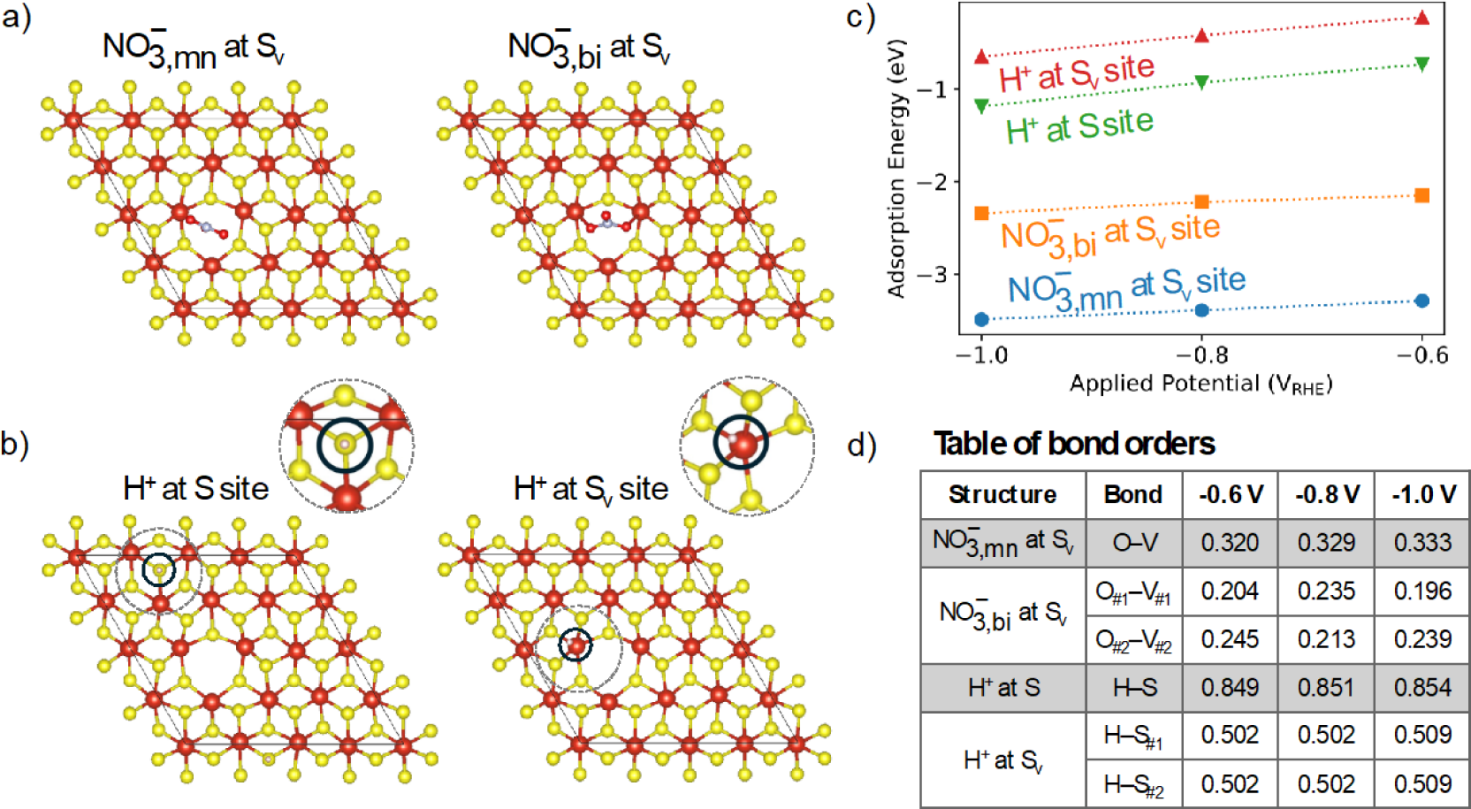
Ball and stick models of VS_2_ (1T-phase) with sulfur vacancy (S_v_) showing optimized structures for (a) NO_3_^–^ adsorption to S_v_ site in a monodentate (NO_3,mn_^–^) and bidentate (NO_3,bi_^–^) configuration and (b) H^+^ adsorption to the S and S_v_ sites (circled and shown as a zoomed in inset for clarity). (c) Calculated NO_3_^–^ and H^+^ adsorption energies at S_v_ as a function of applied potential. (d) Bond orders calculated for all surface-to-adsorbate pairs with bond orders >0.1, most notably showing that the H^+^ is bonded to the two nearest sulfurs and not the vanadium in the H^+^ at S_v_ structure.

To assess the potential for competitive HER, we determined the adsorption energy of a proton at a surface S site and an S_v_ site (converged structures are shown in Figure 4b). Surprisingly, these results show that H^+^ binds favorably at both sites, with similar potential-dependent trends (Figure 4c). To understand these binding characteristics, we performed a charge partitioning analysis using the Density Derived Electrostatic and Chemical Charges version 6 (DDEC6) method.^32^ DDEC6 analysis revealed that the preferred H^+^ binding geometry at the S_v_ site bridges two nearest-neighbor S atoms (with a bond order of 0.5 to each) and is not bonded to the vanadium center (Figure 4d). This observation is consistent with logical expectations, as positively charged protons and negatively charged NO_3_^–^ would be expected to exhibit different binding preferences. Notably, the monodentate NO_3_^–^ binding energy at the S_v_ site is ∼2 eV stronger than that of H^+^ binding across the considered potential range. This suggests that protons are unlikely to compete for the NO_3_RR active sites, although HER may still occur at basal S sites where H^+^ binding is favorable. The difference in binding sites for NO_3_^–^ and H^+^ could be advantageous, as protons are needed for NO_3_RR.

Starting from a monodentate NO_3_^–^ adsorbed at a VS_2_ S_v_ site, we computed the full reaction thermodynamics for three NH_3_-forming NO_3_RR proposed pathways (Figure 5a).^61,62^ These results indicate that all steps in these pathways are thermodynamically favorable at experimentally relevant potentials, with more reductive potentials enhancing the thermodynamic favorability (Figure 5b). While this analysis focuses on NH_3_ formation, it is important to note that several side reactions, beyond HER, can also occur during NO_3_RR.^63,64^ However, a comprehensive analysis of these competing pathways is beyond the scope of this study. Considering the analysis of binding energy and full reaction thermodynamics together, our findings suggest that the activity and selectivity of VS_2_-based electrocatalysts could be tuned by controlling the S_v_ density, with the NO_3_RR being thermodynamically favorable at S_v_ sites. The electrochemical characterization of VS_*x*_ in the previous section (Figure 3a–c) shows higher selectivity toward NO_3_RR compared with VS_2_. This favorable selectivity may suggest the presence of under-coordinated V sites on the surface of VS_*x*_, which would be consistent with theoretical results.

**Figure 5:**
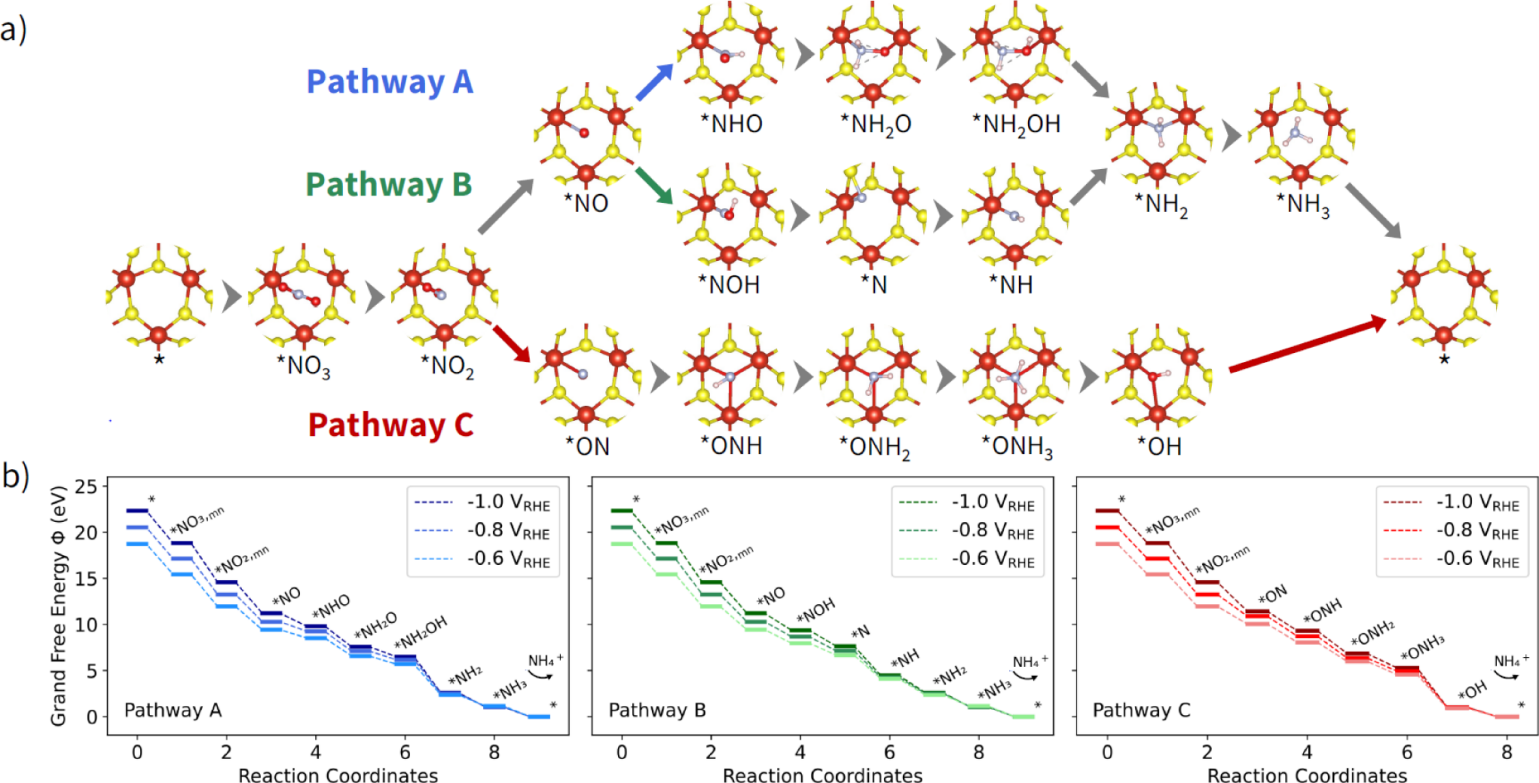
Reaction pathways for the NO_3_RR at a 1T-VS_2_ S_v_ site starting from monodentate NO_3_^–^ (*NO_3_ once adsorbed). (a) Top view of calculated intermediate adsorption structures for three possible NO_3_RR pathways (representative images shown for –0.6 V_RHE_). (b) Corresponding grand free energy reaction profiles for NH_4_^+^ formation along the NO_3_RR reaction pathways under applied potentials.

### Experimental Determination of Kinetic Rate-Limiting Step of NO_3_RR on VS_*x*_

To gain an understanding of the NO_3_RR mechanism on VS_*x*_ experimentally, a KIE experiment is carried out. A KIE is observed if a proton transfer is involved in the kinetic rate-limiting step of a reaction.^65^ When proton transfer is rate-limiting, proton-coupled reactions are expected to be more kinetically facile than deuteron-coupled reactions $\left(j_{H_{2}O}/j_{D_{2}O}>1\right)$ .

For the NO_3_RR, KIE is above unity for CoP (1.5–2.0) in base^66^ as well as Pd (2.06) and Ru (2.74) in base,^67^ suggesting the kinetic rate-limiting step is proton-coupled in these cases. In another example, NO_3_RR on a PdCuCo alloy in neutral pH electrolyte showed a KIE of 1.09, suggesting a kinetic rate-limiting step that is not proton-coupled.^68^ Early work by Koper and coworkers^69^ suggests the first electron transfer to quasi-equilibrated adsorbed NO_3_^–^ (NO_3_*) to be rate-limiting based on Tafel slope and rate order information obtained on noble and coinage metals (Pt, Ir, Ru, Rh, Ag, and Cu), in this case, KIE = 1 would be anticipated. Contrarily, more recent theoretical mechanistic studies—unable to distinguish between nonconcerted proton- and electron-transfer steps—suggest dissociation of NO_3_* (NO_3_* + * → NO_2_* + O*) and rapid hydrogenation of resultant O* as rate-limiting,^63,70^ where a KIE ≠ 1 would be anticipated. In an effort to identify a rate-limiting step on VS_*x*_, we next considered KIE across a range of applied potentials.

The KIE experiments here measure the global effect of switching from H_2_O- to D_2_O-based electrolyte on all Faradaic reactions, with current corresponding to both NO_3_RR and HER. The VS_*x*_ LSVs presented in Figure 6a are qualitatively similar but exhibit differences in magnitude and potential of phosphate mass-transfer limitations. A significant difference is seen between the NO_3_^–^-absent and NO_3_^–^-present electrolyte conditions. In the NO_3_^–^-absent conditions, HER dominates, and KIE is greater than unity at all tested potentials (Figure 6b). In the presence of NO_3_^–^, the KIE hovers near unity across the range of potentials considered, dipping slightly below unity at −0.6 V_RHE_. The KIE-below-unity region in Figure 6b corresponds to the region in Figure 6a where phosphate becomes mass-transfer limited. Stepped chronoamperometry data show behavior similar to that of the LSV (Figure 6c, derived from data in Figure S18).

**Figure 6:**
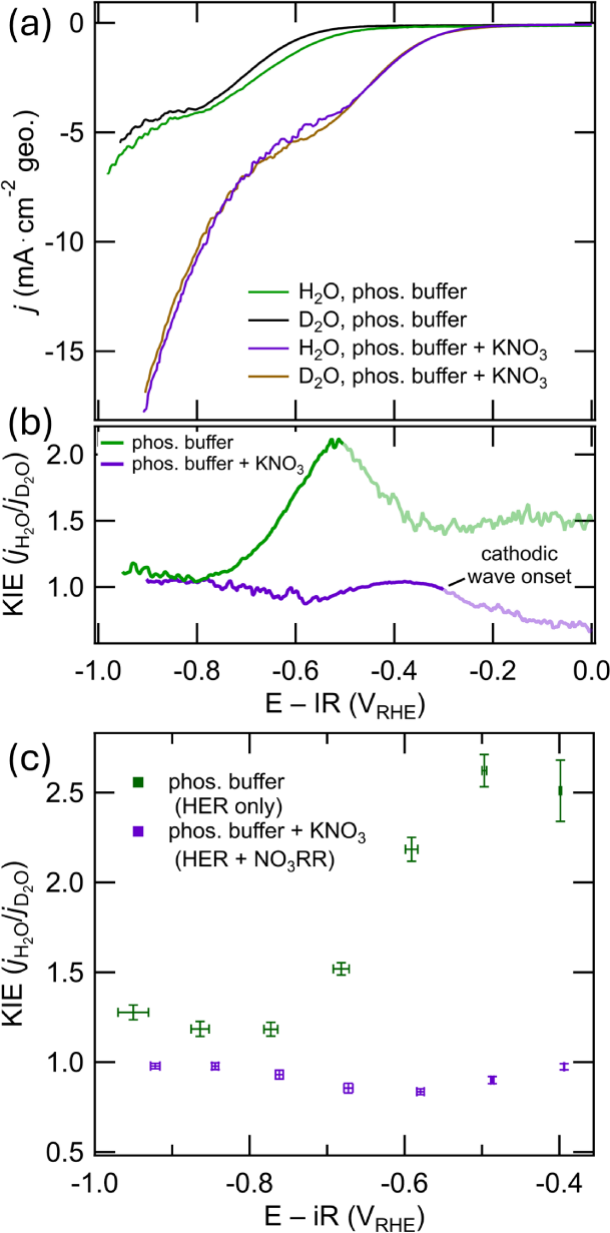
(a) LSVs of VS_*x*_ in H_2_O and D_2_O-based electrolyte (pH 7.0, 0.1 M phosphate buffer) with or without 0.1 M KNO_3_, with forced convection; (b) kinetic isotope effect (KIE, $j_{H_{2}O}/j_{D_{2}O}$ ) calculated from the data in (a); and (c) KIE calculated from stepped chronoamperometry measurements with a 120 s step duration. Error bars in (c) represent combined variation in current (vertical axis and standard deviation) and working electrode potential (horizontal axis and standard deviation) during each potential step for the H_2_O- and D_2_O-based electrolyte measurements.

Considering the KIE close to unity for the NO_3_^–^-present electrolytes in the kinetically limited region with low contribution from HER (for example, at −0.4 V_RHE_), this analysis reveals that the kinetic rate-limiting step of VS_*x*_-catalyzed NO_3_RR at −0.4 V_RHE_ is likely not proton-coupled. At more cathodic potentials, the HER side reaction is convoluted with NO_3_RR, but helpfully, the FE to NH_4_^+^ across these potentials (Figure 3b) is high and fairly constant. The KIE close to unity in the range of −0.5 to −1.0 V_RHE_ suggests that the kinetic rate-limiting step of the NO_3_RR in this potential region is also likely not proton-coupled. This analysis suggests that a nonproton-coupled first electron transfer to adsorbed NO_3_* is the kinetic rate-limiting step of NO_3_RR on VS_*x*_; however, additional studies are necessary to confirm this. The theoretical analysis of the NO_3_RR on the VS_2_ S_v_ site described in the preceding section assessed energy differences between the intermediate products of the NO_3_RR via several pathways. At the potentials considered in the theoretical analysis, a potential-determining step was not observed, as each reaction step was thermodynamically downhill, resulting in a lower-energy product. However, kinetic rate-limiting steps were not explicitly evaluated since transition states were not calculated.

## Conclusions

A polycrystalline vanadium sulfide catalyst, VS_*x*_, is synthesized by annealing solvothermally grown VS_2_ under inert atmosphere and demonstrates improved selectivity and activity toward NO_3_RR in phosphate-buffered neutral pH electrolyte. Characterization by microscopy (SEM, TEM) shows little morphological difference between VS_*x*_ and VS_2_, while diffraction methods (XRD and SAED) show increased crystallinity of VS_*x*_ in comparison with VS_2_ but do not present a clear indication of the phase of VS_*x*_. Characterization by spectroscopy methods (NEXAFS and XPS) indicates a modified S and V environment in VS_*x*_ in comparison to VS_2_, as well as lower relative oxide character.

The VS_*x*_ outperforms VS_2_ in electrochemical measurements of selectivity and activity toward NO_3_RR, with a maximum activity of 2.3 ± 0.6 mg·cm^–2^ geo.·h^–1^ @ −0.92 V_RHE_ and maximum FE to NH_4_^+^ of 69 ± 6% @ −0.69 V_RHE_ in the presence of 0.1 M NO_3_^–^. The annealing step reduces the oxide character of VS_*x*_ compared to VS_2_ and introduces a modified local V bonding environment, resulting in improved NO_3_RR activity, likely due to the creation of active sites for NO_3_^–^ binding. The NO_3_RR mechanism is investigated through GC-DFT simulations and KIE measurements. The DFT calculations indicate that undercoordinated vanadium sites provide favorable binding sites for NO_3_^–^ over H^+^. Further, DFT calculations show that in several tested reaction mechanisms, all reaction steps are exergonic, suggesting that the formation of S_v_ sites in vanadium sulfides could lead to improved NO_3_RR activity in VS_*x*_. However, the increased relative crystallinity and decreased relative oxide character of VS_*x*_ versus VS_2_ cannot be ruled out as contributing factors. KIE measurements suggest that the kinetic rate-limiting step of NO_3_RR on VS_*x*_ in phosphate-buffered neutral pH electrolyte is not proton-coupled, and thus may be the first electron transfer to adsorbed NO_3_*, adding to the ongoing discussion of mechanistic rate-limiting steps for the complex NO_3_RR. Taken together, the results indicate that the improved NO_3_RR performance of VS_*x*_ likely arises from a combination of factors introduced by the annealing process, including modified local bonding environments, increased crystallinity, and reduced surface oxidation.

## Supplementary Material



## Notes

Previously reported vanadium-containing NO_3_RR catalyst composition and experimental conditions, which resulted in highest reported Faradaic efficiency in electrolyte with NO_3_^–^ ion concentration ≤0.1 M (Table S1); lattice spacings in VS_2_ and VS_*x*_ determined by selected area electron diffraction (SAED) measurements of multiple areas within each surveyed sample (Table S2); stoichiometry of VS_2_ and VS_*x*_ as assessed by energy dispersive spectroscopy (EDS) and X-ray photoelectron spectroscopy (XPS) methods (Table S3); fitted peak positions for V L_3_-edge total electron yield near edge X-ray absorption fine structure (TEY NEXAFS) spectra shown in Figures 2 and S8. (Table S4); XPS Peak positions and shifts (Table S5); electrochemical control experiments (Table S6); SEM images comparing the bare carbon paper substrate (top) with VS_2_ (middle) and VS_*x*_ (bottom) (Figure S1); SEM image of a cross section of VS_2_ (left) with enlarged area (right) showing catalyst layer cross section (Figure S2); TEM image of VS_*x*_ showing single-crystal domains and corresponding fast Fourier transform (FFT) plot showing spots corresponding to lattice plane spacings within the TEM image (Figure S3); lattice spacings of (a) VS_2_ and (b) VS_*x*_ as measured by selected area electron diffraction (SAED) measurements (red and blue circles), as well as calculated lattice spacings of several vanadium sulfide and vanadium oxide species (black squares) (Figure S4); Discussion of SAED-measured lattice spacings; STEM-EDS map of VS_*x*_. (a) Annular dark-field (ADF) STEM image of VS*_x_* and (b–e) simultaneously acquired elemental maps showing colocation of vanadium and sulfur, with a low concentration of oxygen (Figure S5); XRD patterns of VS_2_ and VS_*x*_ grown on a carbon paper substrate, along with the carbon paper substrate (Figure S6; discussion of X-ray diffraction (XRD) results; comparison of NEXAFS surface sensitive (∼5 nm) total electron yield (TEY) and bulk-sensitive (>100 nm) total fluorescence yield (TFY) detection modes for VS_2_ and VS_*x*_ (Figure S7); Gaussian fittings of NEXAFS data presented in Figure 2a of the main text for (a) single-crystal VS_2_ (reproduced with permission from Mulazzi et al.), (b) V_2_O_5_, (c) V_2_O_3_, (d) VS_2_, and (e) VS_*x*_. (Figure S8); (a) XPS N1s spectra of VS_2_ and VS_*x*_, (b) V2p spectra of vanadium oxide references, (c) O1s spectra of VS_2_, VS_*x*_, and vanadium oxide references, and (d–g) XPS spectra from main text Figure 2(b–e) with extended electron binding energy range (Figure S9); comparison of VSx LSVs with stirring (dashed line) and without stirring (solid line) (Figure S10); (a) LSV characterization of VS_2_, VS_*x*_, and carbon paper substrate data from Main Text Figure 3 with surface area reported as electrochemically active surface area, and (b) activity characterization of VS_2_, VSx, and carbon paper substrate data from main text Figure 3 with surface area reported as electrochemically active surface area (Figure S11); representative double layer capacitance measurements for determination of electrochemically active surface area (ECSA) of (a) carbon paper, (b) VS2, and (c) VS*_x_* (0.1 M phosphate buffer (pH 7.0)) (Figure S12); explanation of ECSA calculation; chronoamperometry traces of VS_2_ (left) and VS*_x_* (right) during bulk electrolysis experiments in phosphate-buffered (pH 7.0, 0.1 M) KNO_3_ (0.1 M) electrolyte (Figure S13); estimate of NO_3_^–^ remaining upon completion of bulk electrolysis experiments described in the main text (Figure S14); explanation of calculation of remaining NO_3_^–^ upon completion of bulk electrolysis experiments; SEM images of post-electrocatalysis VS_2_ (top) and VS*_x_* (middle) at the most cathodic potentials tested. The VS_2_ shows significant morphology changes, while the VS*_x_* does not show significant morphology changes. Also shown is VS*_x_* after a 6 h, *j* = 30 mA·cm^–2^geo. durability test (bottom), which shows some evidence of flaking. (Figure S15); VS*_x_* NO3RR selectivity and activity concentration dependence at nominal *E* = 0.9 V_RHE_ with 85% active IR correction (Figure S16); (a, b) views of models of fully coordinated 1T-phase VS_2_ surface structures where NO_3_^–^ does not adsorb (unconverged). (c, d) Edge model of 1T-phase VS_2_ with NO_3_^–^ adsorbed in monodentate (c) and bidentate (d) configurations. Structures shown are converged at −0.6 VRHE. (e) Calculated NO_3_^–^ adsorption energies at VS_2_ edge, showing overly favorable binding energies (Figure S17); stepped chronoamperometry of VS*_x_* in H_2_O and D_2_O-based electrolyte (pH 7.0, 0.1 M phosphate buffer), with or without 0.1 M KNO_3_, with forced convection (Figure S18) (PDF)
