# Supplementary material for: Nitrate-to-Ammonia Electroconversion at Neutral pH on Polycrystalline Vanadium Sulfide Derived from Vanadium Disulfide
Source: ACS Appl Energy Mater. 2025 Jun 16;8(13):9407–18. doi: 10.1021/acsaem.5c01047 (PMC12264862; doi:10.1021/acsaem.5c01047)
Supplement: Supplementary file 1 [file ae5c01047_si_001.pdf]

Supporting Information

Nitrate to Ammonia Electroconversion at Neutral pH on Polycrystalline Vanadium Sulfide Derived from Vanadium Disulfide

Logan M. Wilder[1], Taylor J. Aubry[1], Carter S. Gerke[2], O. Quinn Carvalho[1], Jonathan R. Thurston[3], Michael F. Toney[1,4,5], Michelle A. Smeaton[1], Jao van de Lagemaat[1,5], Elisa M. Miller[1,5*]

[1]Materials, Chemical, and Computational Science, National Renewable Energy Laboratory, 15013 Denver W Pkwy, Golden, CO 80401, USA

[2]Department of Chemistry, Johns Hopkins University, Baltimore, MD 21218, USA

[3]Department of Chemistry, University of Colorado Boulder, Boulder, CO 80309, USA

[4]Department of Chemical and Biological Engineering, University of Colorado Boulder, Boulder, CO 80309, USA

[5]Materials Science and Engineering Program, Renewable and Sustainable Energy Institute, Boulder, CO 80303, USA

*Corresponding author: Elisa.Miller@nrel.gov

Table of Contents

	Page
Table S1. Previously Reported Vanadium-Containing NO_3RR Catalyst Composition and Experimental Conditions Which Resulted in Highest Reported Faradaic Efficiency in Electrolyte With NO_3^- Ion Concentration ≤ 0.1 M	S4
Table S2. Lattice Spacings in VS_2 and VS_x Determined by Selected Area Electron Diffraction (SAED) Measurements of Multiple Areas Within Each Surveyed Sample	S5
Table S3. Stoichiometry of VS_2 and VS_x as Assessed by Energy Dispersive Spectroscopy (EDS) and X-ray Photoelectron Spectroscopy (XPS) Methods	S6
Table S4. Fitted Peak Positions for V L_3-edge Total Electron Yield Near Edge X-ray Absorption Fine Structure (TEY NEXAFS) Spectra Shown in Figure 2 and Figure S8.	S7
Table S5. XPS Peak Positions and Shifts	S8
Table S6. Electrochemical Control Experiments	S9
Figure S1. SEM images comparing the bare carbon paper substrate (top) with VS_2 (middle) and VS_x (bottom).	S10
Figure S2. SEM image of a cross section of VS_2 (left) with enlarged area (right) showing catalyst layer cross section.	S11
Figure S3. TEM image of VS_x showing single-crystal domains and corresponding fast Fourier transform (FFT) plot showing spots corresponding to lattice plane spacings within the TEM image.	S12
Figure S4. Lattice spacings of (a) VS_2 and (b) VS_x as measured by selected area electron diffraction (SAED) measurements (red and blue circles), as well as calculated lattice spacings of several vanadium sulfide and vanadium oxide species (black squares).	S13
Discussion of SAED-measured Lattice Spacings	S13-S14
Figure S5. STEM-EDS map of VS_x. (a) Annular dark-field (ADF) STEM image of VSx and (b-e) simultaneously acquired elemental maps showing co-location of vanadium and sulfur, with a low concentration of oxygen.	S15
Figure S6. XRD patterns of VS_2 and VS_x grown on a carbon paper substrate, along with the carbon paper substrate.	S16
Discussion of X-ray Diffraction (XRD) Results	S17
Figure S7. Comparison of NEXAFS surface sensitive (□5 nm) total electron yield (TEY) and bulk-sensitive (>100 nm) total fluorescence yield (TFY) detection modes for VS_2 and VS_x.	S18
Figure S8. Gaussian fittings of NEXAFS data presented in Figure 2(a) of the Main Text for (a) single crystal VS_2 (reproduced with permission from Mulazzi *et al.*)[1], (b) V_2O_5, (c) V_2O_3, (d) VS_2 and (e) VS_x.	S19

Figure S9. (a) XPS N1s spectra of VS_2 and VS_x, (b) V2p spectra of vanadium oxide references, (c) O1s spectra of VS_2, VS_x, and vanadium oxide references, and (d-g), XPS spectra from Main Text Figure 2(b-e) with extended electron binding energy range.	S20
Figure S10. Comparison of VS_x LSVs with stirring (dashed line) and without stirring (solid line).	S21
Figure S11. (a) LSV characterization of VS_2, VS_x, and carbon paper substrate data from Main Text Figure 3 with surface area reported as electrochemically active surface area and (b), activity characterization of VS_2, VS_x, and carbon paper substrate data from Main Text Figure 3 with surface area reported as electrochemically active surface area.	S22
Figure S12. Representative double layer capacitance measurements for determination of electrochemically active surface area (ECSA) of (a) carbon paper, (b) VS_2, and (c) VS_x (0.1 M phosphate buffer (pH 7.0)).	S23
Explanation of ECSA Calculation	S23-S24
Figure S13. Chronoamperometry traces of VS_2 (left) and VS_x (right) during bulk electrolysis experiments in phosphate-buffered (pH 7.0, 0.1 M) KNO_3 (0.1 M) electrolyte.	S25
Figure S14. Estimate of NO_3^- remaining upon completion of bulk electrolysis experiments described in the Main Text.	S26
Explanation of Calculation of Remaining NO3– Upon Completion Of Bulk Electrolysis Experiments	S26
Figure S15. SEM images of post-electrocatalysis VS_2 (top) and VS_x (middle) at the most cathodic potentials tested. The VS_2 shows significant morphology changes, while the VS_x does not show significant morphology changes. Also shown is VS_x after a 6 h, j = 30 $mA\cdot cm^{-2}$geo. durability test (bottom) which shows some evidence of flaking.	S27
Figure S16. VS_x NO_3RR selectivity and activity concentration dependance at nominal E = 0.9 V_{RHE} with 85% active IR correction.	S28
Figure S17. (a-b) Views of models of fully coordinated 1T-phase VS_2 surface structures where NO_3^- does not adsorb (unconverged). (c-d) Edge model of 1T-phase VS_2 with NO_3^- adsorbed in monodentate (c) and bidentate (d) configurations. Structures shown are converged at -0.6 V_{RHE}. (e) Calculated NO_3^- adsorption energies at VS_2 edge, showing overly favorable binding energies.	S29
Figure S18. Stepped chronoamperometry of VS_x in H_2O and D_2O-based electrolyte (pH 7.0, 0.1 M phosphate buffer), with or without 0.1 M KNO_3, with forced convection.	S30
Supporting Information References	S31

Table S1. Previously Reported Vanadium-Containing NO_3RR Catalyst Composition and Experimental Conditions Which Resulted in Highest Reported Faradaic Efficiency in Electrolyte With NO_3^- Ion Concentration ≤ 0.1 M

Catalyst	Electrolyte	Highest reported NH_3 or NH_4^+ yield rate	Highest reported Faradaic efficiency to NH_3 or NH_4^+	Cit.
V-doped MoS_2	1.613 mM (100 ppm) $NaNO_3^-$ + 0.5 M Na_2SO_4	232 μg·h^{-1}·mgcat.$^{-1}$ @ –1.1 V_{RHE}	not reported	[2]
VO_x, mix of V^{3+} & V^{4+}	0.1 M $NaNO_3$ adjusted to pH 3.2 with HNO_3	890 μmole·h^{-1}·gcat.$^{-1}$ @ –0.464 V_{RHE}	25% @ –0.464 V_{RHE}	[3]
VO_{2-x}/CuF	0.1 M KNO_3 + 0.1 M K_2SO_4	1.833 mmol·h^{-1}·mgcat.$^{-1}$ @ –1.3 V_{RHE}	77.9% @ –1.3 V_{RHE}	[4]
La-doped VS_{2-x}	0.1 M KNO_3 + 0.5 M Na_2SO_4	13.5 mg·cm^{-2} geo.·h^{-1} @ –0.7 V_{RHE}	96.6% @ –0.6 V_{RHE}	[5]
VS_2[a]	0.1 M KNO_3 + 0.5 M Na_2SO_4	5.9 mg·cm^{-2} geo.·h^{-1} @ –0.6 V_{RHE} [a]	68.6% @ –0.6 V_{RHE} [a]	[5]
VS_2	0.1 M KNO_3 + 0.1 M phos. Buffer pH 7.0	0.5 ± 0.1 mg·cm^{-2} geo.·h^{-1} @ –0.95 V_{RHE}	43 ± 3% @ –0.59 V_{RHE}	*this work*
VS_x	0.1 M KNO_3 + 0.1 M phos. Buffer pH 7.0	2.3 ± 0.6 mg·cm^{-2} geo.·h^{-1} @ –0.92 V_{RHE}	69 ± 6% @ –0.69 V_{RHE}	*this work*

[a]values for VS_2 reported by Chu and coworkers[5] were extracted from a visual representation of the data.

Table S2. Lattice Spacings in VS_2 and VS_x Determined by Selected Area Electron Diffraction (SAED) Measurements of Multiple Areas Within Each Surveyed Sample

VS_2			VS_x		
**Lattice spacing (Å)**	Type of feature	VS_2 area sampled	**Lattice spacing (Å)**	Type of feature	VS_x area sampled
**2.87**	spot	Area 2	**2.90**	ring	Area 2
**2.76**	spot and ring	Area 3	**2.87**	ring	Area 1
**2.75**	diffuse ring	Area 2	**2.60**	ring	Area 2
**2.59**	diffuse ring	Area 1	**2.60**	ring	Area 4
**2.06**	spot	Area 1	**2.58**	ring	Area 1
**2.03**	spot	Area 2	**2.57**	ring	Area 3
**1.99**	spot	Area 2	**2.14**	ring	Area 4
**1.64**	ring	Area 2	**2.13**	diffuse ring	Area 1
**1.64**	ring	Area 1	**2.10**	ring	Area 3
**1.64**	ring	Area 3	**2.02**	ring	Area 2
**1.59**	ring	Area 3	**1.93**	ring	Area 2
**1.55**	spot	Area 1	**1.68**	ring	Area 1
**1.40**	faint ring	Area 1	**1.68**	ring	Area 2
**1.39**	faint ring	Area 2	**1.68**	ring	Area 3
**1.23**	spot	Area 1	**1.67**	ring	Area 4
**1.13**	diffuse ring	Area 3	**1.65**	ring	Area 4
**1.05**	faint ring	Area 1	**1.64**	ring	Area 2
**0.99**	spot	Area 1	**1.64**	ring	Area 1
			**1.64**	ring	Area 3
			**1.40**	faint ring	Area 2
			**1.39**	faint ring	Area 1
			**1.29**	faint ring	Area 1
			**1.24**	ring	Area 3
			**1.24**	ring	Area 4
			**1.17**	ring	Area 4
			**1.14**	diffuse ring	Area 2
			**1.07**	ring	Area 4

Table S3. Stoichiometry of VS_2 and VS_x as Assessed by Energy Dispersive Spectroscopy (EDS) and X-ray Photoelectron Spectroscopy (XPS) Methods

	S:V	S:V
Method	VS_2	VS_x
SEM EDS[a,b]	1.5	1.0
TEM EDS[a,b]	1.6	1.3
XPS[c,b]	1.9	2.0
XPS VS_x component only[c,d]	2.6	2.5

[a]bulk-sensitive method

[b]includes V cont. from VO_x

[c]surface-sensitive method

[d]excludes V cont. from VO_x

Discussion of VS_2 and VS_x Stoichiometry as Measured by EDS (Bulk Sensitive) and XPS (Surface Sensitive) Methods

Analysis of the bulk stoichiometry of VS_2 and VS_x by SEM EDS and TEM EDS show a reduction in the bulk S content of the annealed VS_x in comparison to VS_2, shown in Table S3. Analysis of the surface stoichiometry by XPS does not show a significant change in surface S:V ratio. Both VS_2 and VS_x show some VO_x character as revealed by XPS and NEXAFS, and the XPS ratio of VS (all stoichiometries) to VO (all stoichiometries) shows the VS:VO ratio is increased during the annealing step (2.8 for VS_2 and 4.4 for VS_x).

Table S4. Fitted Peak Positions for V L_3-edge Total Electron Yield Near Edge X-ray Absorption Fine Structure (TEY NEXAFS) Spectra Shown in Figure 2 and Figure S8.

	Peak Number	Peak center (eV)	Amplitude (a.u.)	FWHM (eV)	Percent area (%)
**V_2O_3**	1	515.2	1.37	2.00	12.67
	2	516.8	3.76	1.92	34.81
	3	518.3	3.73	1.69	34.52
	4	520.2	1.94	1.99	18.00
**V_2O_5**	1	516.6	1.76	1.99	26.45
	2	518.3	3.72	1.71	55.74
	3	520.4	1.19	2.00	17.81
**VS_2 single crystal[a]**	1	514.4	0.83	2.00	23.47
	2	516.2	1.38	1.53	38.90
	3	517.7	0.80	1.99	22.51
	4	520.0	0.54	2.00	15.12
**VS_2**	1	514.5	2.24	2.11	12.72
	2	516.5	6.71	2.08	38.05
	3	518.3	5.26	1.89	29.86
	4	520.5	3.41	2.12	19.37
**VS_x**	1	514.6	5.00	2.20	23.04
	2	516.2	8.00	1.93	36.83
	3	517.9	3.94	1.93	18.15
	4	520.2	4.77	2.50	21.98

[a]VS_2 single crystal data is reproduced with permission from Mulazzi *et al.*[1]

Table S5. XPS Peak Positions and Shifts

	VS_2	VS_x	
S character	Peak position (eV)	Peak position (eV)	VS_2-VS_x position (eV)
S^{2-}	160.9	160.7	0.2
S_2^{2-}	161.9	161.5	0.4
(S^{2-}) - (S_2^{2-})	-1.1	-0.9	
	Area ratios	Area ratios	
$(S^{2-}):(S_2^{2-})$	1.8	0.2	
V character	Peak position (eV)	Peak position (eV)	VS_2-VS_x position (eV)
V-S	513.2	512.7	0.5

Table S6. Electrochemical Control Experiments

Control experiment	Electrode	Electrolyte	E –iR (V_{RHE})	activity	Faradaic efficiency to NH_4^+
**No-NO_3^- control**	VS_2	0.1 M potassium phosphate buffer (pH 7.0)	–0.95	0.0088 mg·cm^{-2} geo.·h^{-1} [a]	-
**No-NO_3^- control**	VS_x	0.1 M potassium phosphate buffer (pH 7.0)	–0.96	0.0093 mg·cm^{-2} geo.·h^{-1} [a]	-
**No-catalyst control**	Carbon paper substrate only	0.1 M potassium phosphate buffer (pH 7.0) + 0.1 M KNO_3	–0.93	0.22 mg·cm^{-2} geo.·h^{-1}	38%

[a] false positive signal

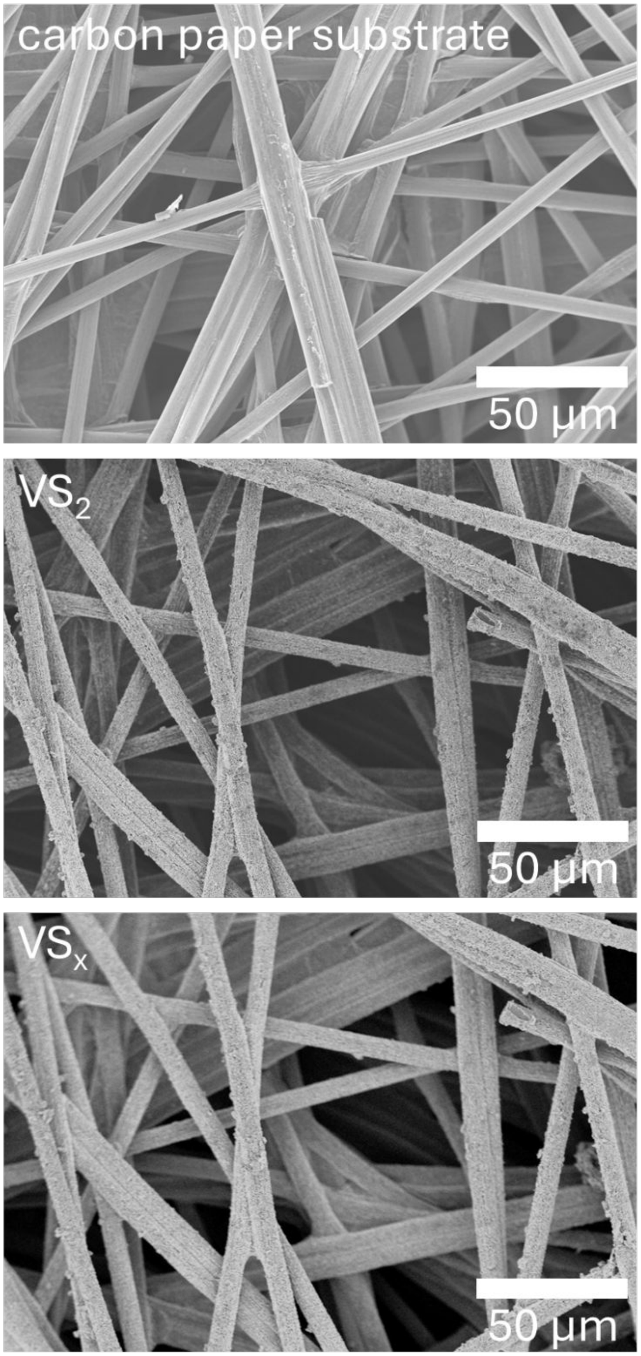

Figure S1. SEM images comparing the bare carbon paper substrate (top) with VS_2 (middle) and VS_x (bottom). The VS_2 and VS_x images were collected by analyzing the same sample in the same location before and after the annealing step.

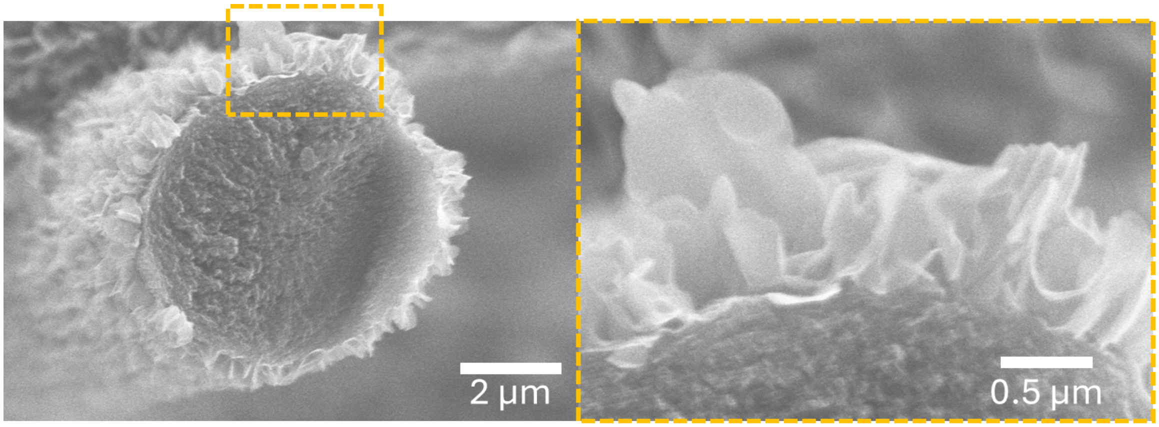

Figure S2. SEM image of a cross section of VS_2 (left) with enlarged area (right) showing catalyst layer cross section.

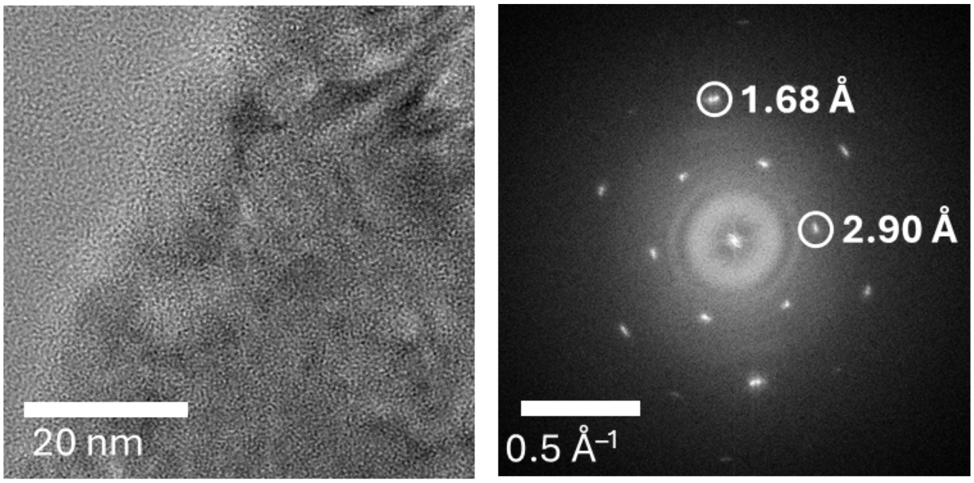

Figure S3. TEM image of VS_x showing single-crystal domains and corresponding fast Fourier transform (FFT) plot showing spots corresponding to lattice plane spacings within the TEM image.

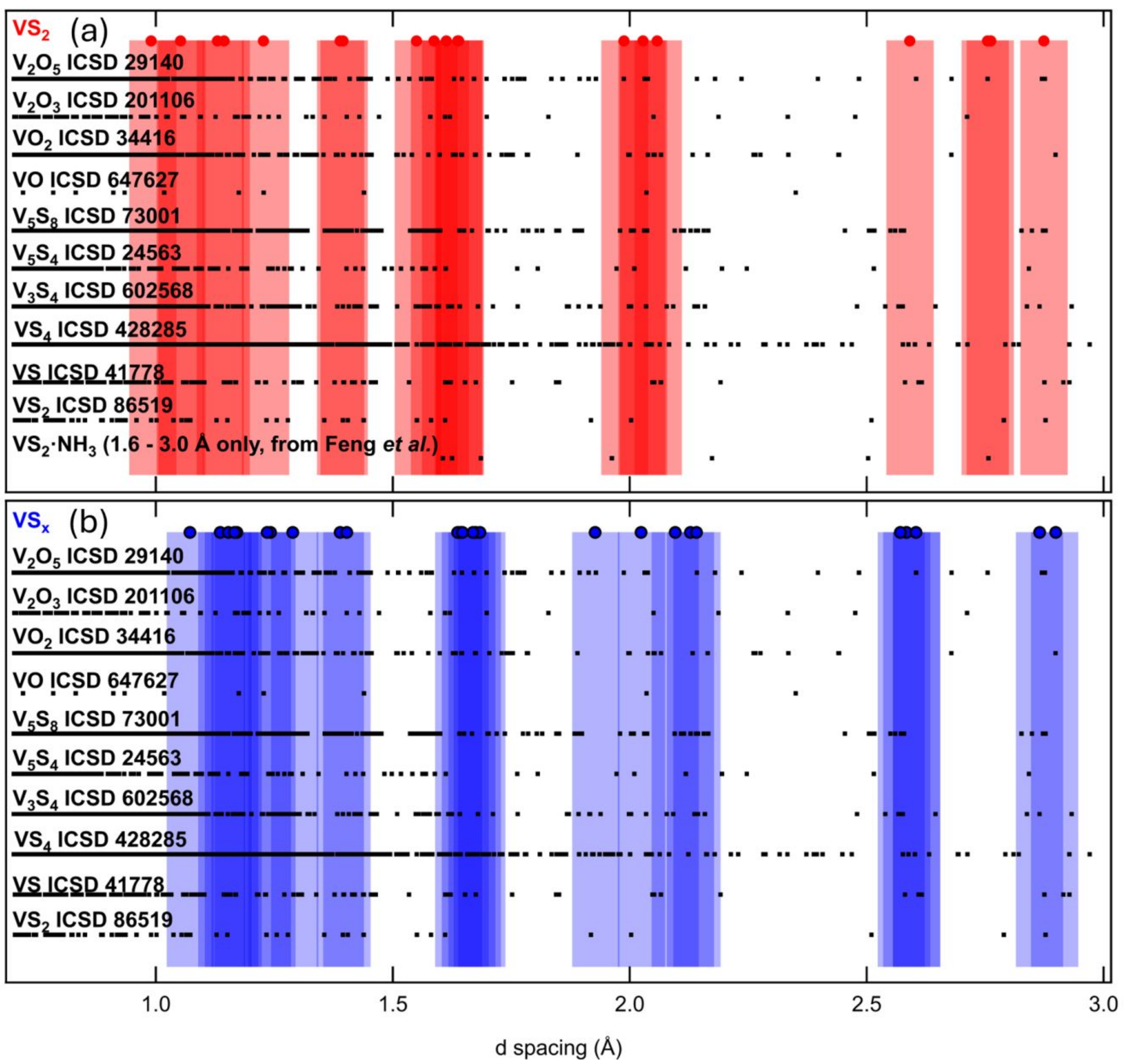

Figure S4. Lattice spacings of (a) VS_2 and (b) VS_x as measured by selected area electron diffraction (SAED) measurements (red or blue circles), as well as calculated lattice spacings of several vanadium sulfide and vanadium oxide species (black squares). The SAED-measured lattice spacings are measured from four areas of each sample and all measured lattice spacings are shown in the figure. The SAED-measured lattice spacings are also shown in Table S2. The $VS_2 \cdot NH_3$ lattice spacings are from Feng *et al.*[6]

Discussion of SAED-measured Lattice Spacings

It is likely that the unannealed, as-synthesized VS_2 contains intercalated small molecules such as NH_3 as well as other intercalated molecules such as octylamine (used in the synthesis of VS_2) which would expand the interlayer spacing of the material. As shown in Figure S4, there is some overlap of the SAED-measured lattice spacings of VS_2 with both VS_2 (ICSD 86519) and NH_3-intercalated VS_2 (reported in Feng *et al.* [6]), as well as several additional vanadium sulfide phases.

Similarly, the SAED-measured lattice spacings of VS_x shown in Figure S4 could be indexed to the lattice spacings of several reported vanadium sulfide phases as shown.

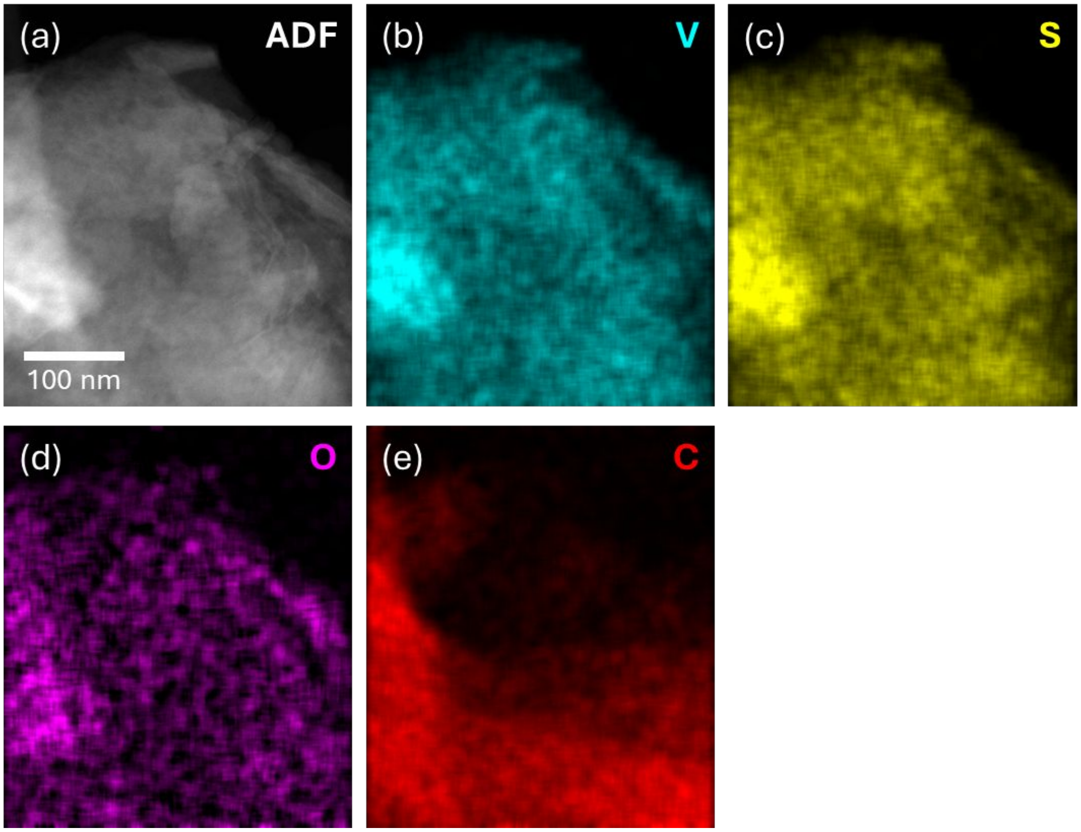

Figure S5. STEM-EDS map of VS_x. (a) Annular dark-field (ADF) STEM image of VS_x and (b-e) simultaneously acquired elemental maps showing co-location of vanadium and sulfur, with a low concentration of oxygen. The carbon signal arises primarily from the lacey carbon support the VS_x is sitting on.

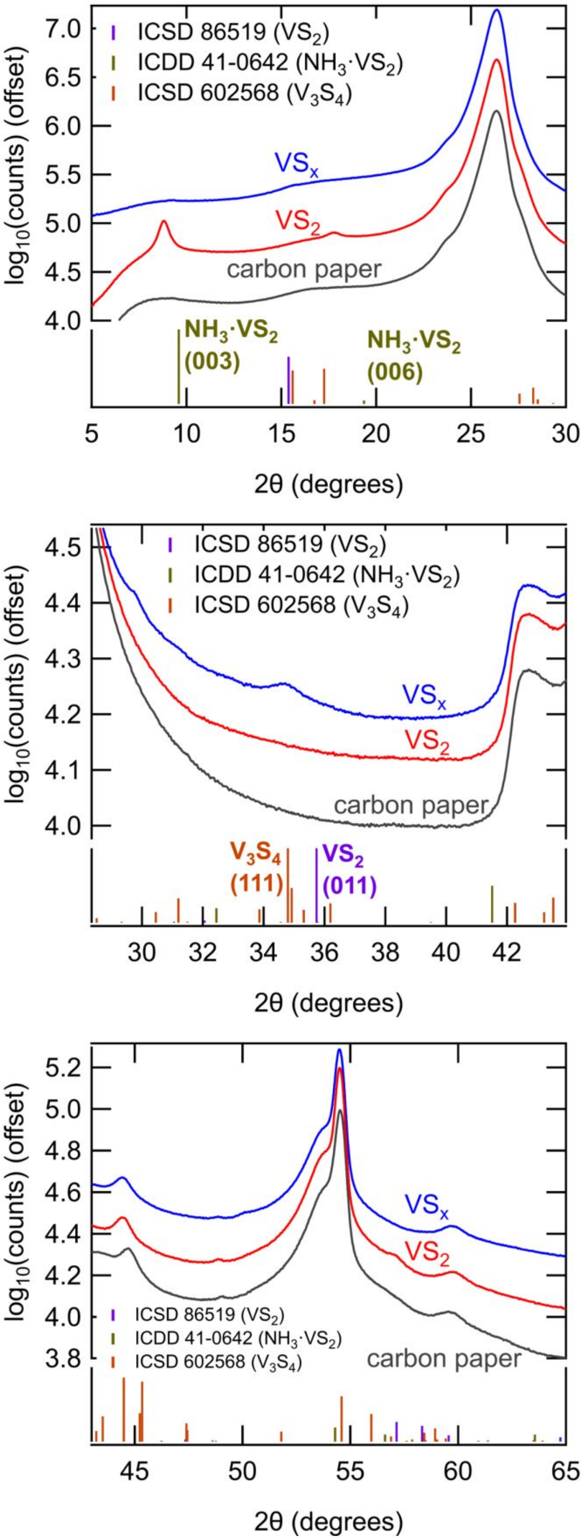

Figure S6. XRD patterns of VS_2 and VS_x grown on a carbon paper substrate, along with the carbon paper substrate. The top, middle and bottom figures represent separate scans of the same samples in different regions of 2θ.

Discussion of X-ray Diffraction (XRD) Results

For XRD characterization of the vanadium sulfide materials, Diffraction patterns are collected with a Bruker D8 Discover equipped with a 2-dimensional area detector (2D-XRD), where 2θ information are integrated over chi along the range of denoted 2θ. The XRD pattern of VS_2 shows two narrow diffraction peaks at 8.8° and 17.8° 2θ which likely correspond to the (003) and (006) planes of interlayer-expanded VS_2, and this shows a similar XRD pattern to NH_3-intercalated VS_2.[6] A similar assignment for the 8.9° and 17.9° 2θ peaks is suggested by Zhang *et al.*[7] as expanded (001) and (002) planes of VS_2, with the interlayer expansion arising from intercalated octylamine, the solvent used during solvothermal synthesis. The most likely case is that a mixture of small molecules including S, N, and C-containing compounds are intercalated into the as-grown VS_2. Evidence for N-containing species in VS_2 which are reduced in VS_x is shown in XPS characterization of these materials (Figure S7), which reveals the N 1s peak (position is consistent with N containing organic matrix) is greatly reduced in VS_x versus VS_2. Other sharp peaks do not appear in the XRD pattern of VS_2, likely indicating a template-driven preferred orientation of the crystallites. After annealing VS_2 to obtain VS_x, the diffraction peaks at 8.9° and 17.9° 2θ disappear and a low-intensity peak at 34.7° 2θ appears. The peak at 34.7° 2θ may correspond to the (011) peak of VS_2 (ICSD 86519). An alternative assignment to the peak at 34.7° 2θ could be the (111) peak of V_3S_4 (ICSD 602568), which should occur at 34.8 2θ, as shown in Figure S6, or, the (111) peak of V_5S_4 (ICSD 73001), which should occur at 35.2 2θ. This analysis suggests that intercalated small molecules are removed from VS_2 during annealing, and that VS_x shows some crystalline VS_2-like, V_3S_4-like, or V_5S_4-like character.

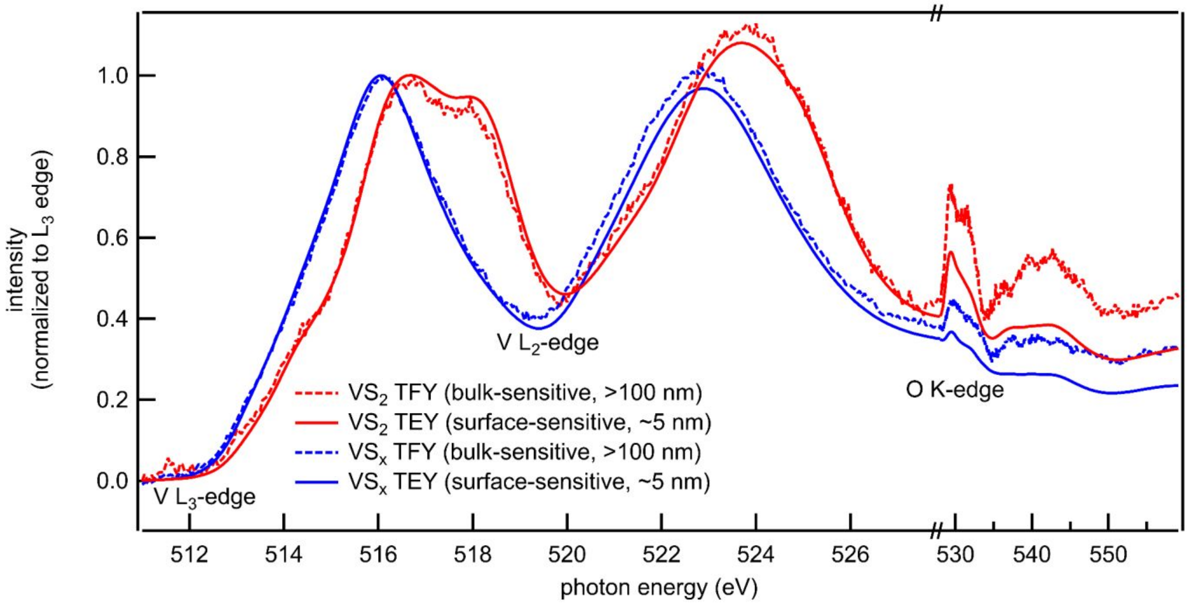

Figure S7. Comparison of NEXAFS surface sensitive (□5 nm) total electron yield (TEY) and bulk-sensitive (>100 nm) total fluorescence yield (TFY) detection modes for VS_2 and VS_x.

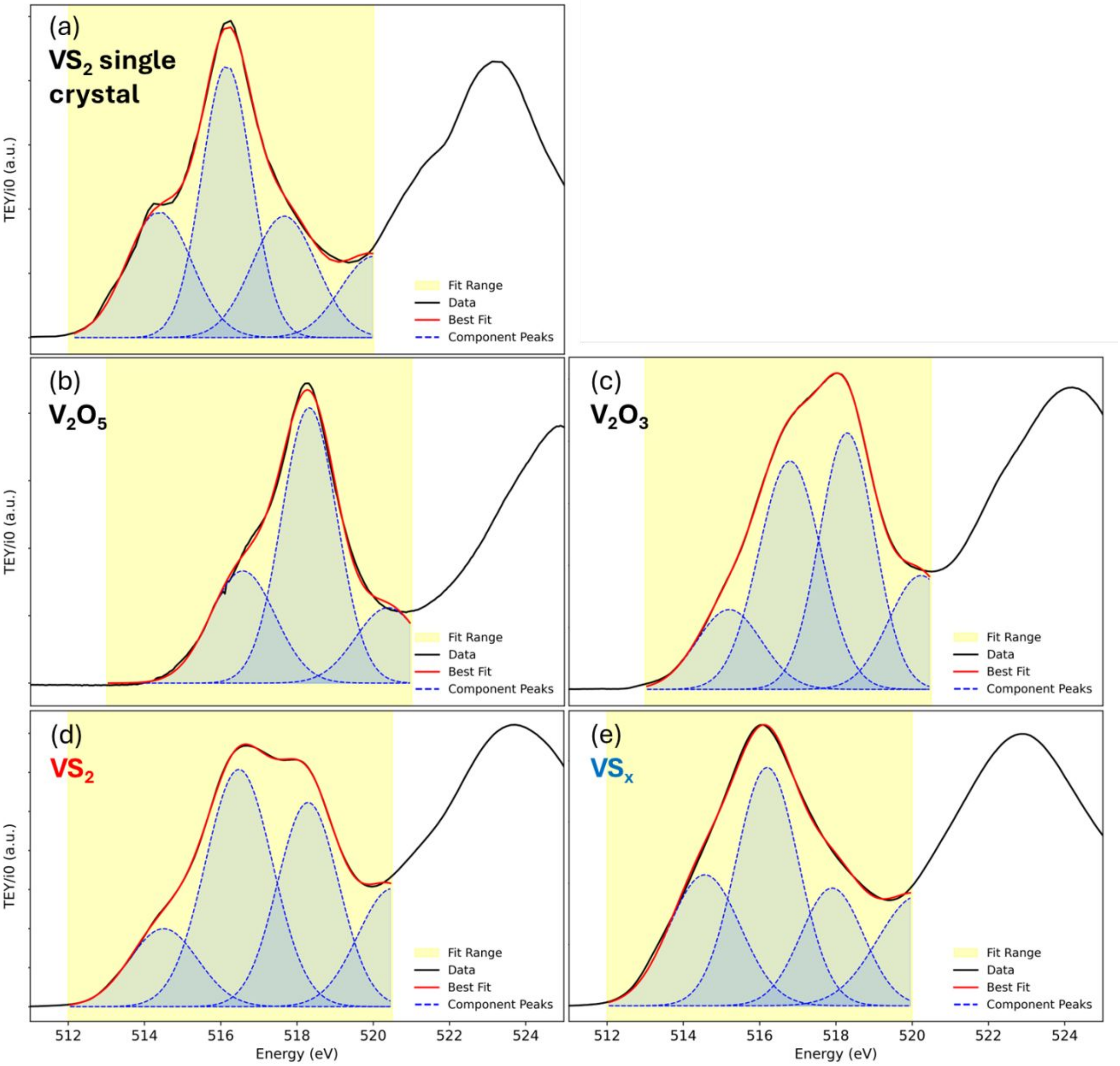

Figure S8. Gaussian fittings of NEXAFS data presented in Figure 2(a) of the Main Text for (a) single crystal VS_2 (reproduced with permission from Mulazzi *et al.*)[1], (b) V_2O_5, (c) V_2O_3, (d) VS_2 and (e) VS_x. The fitting parameters for all materials are provided in Table S1.

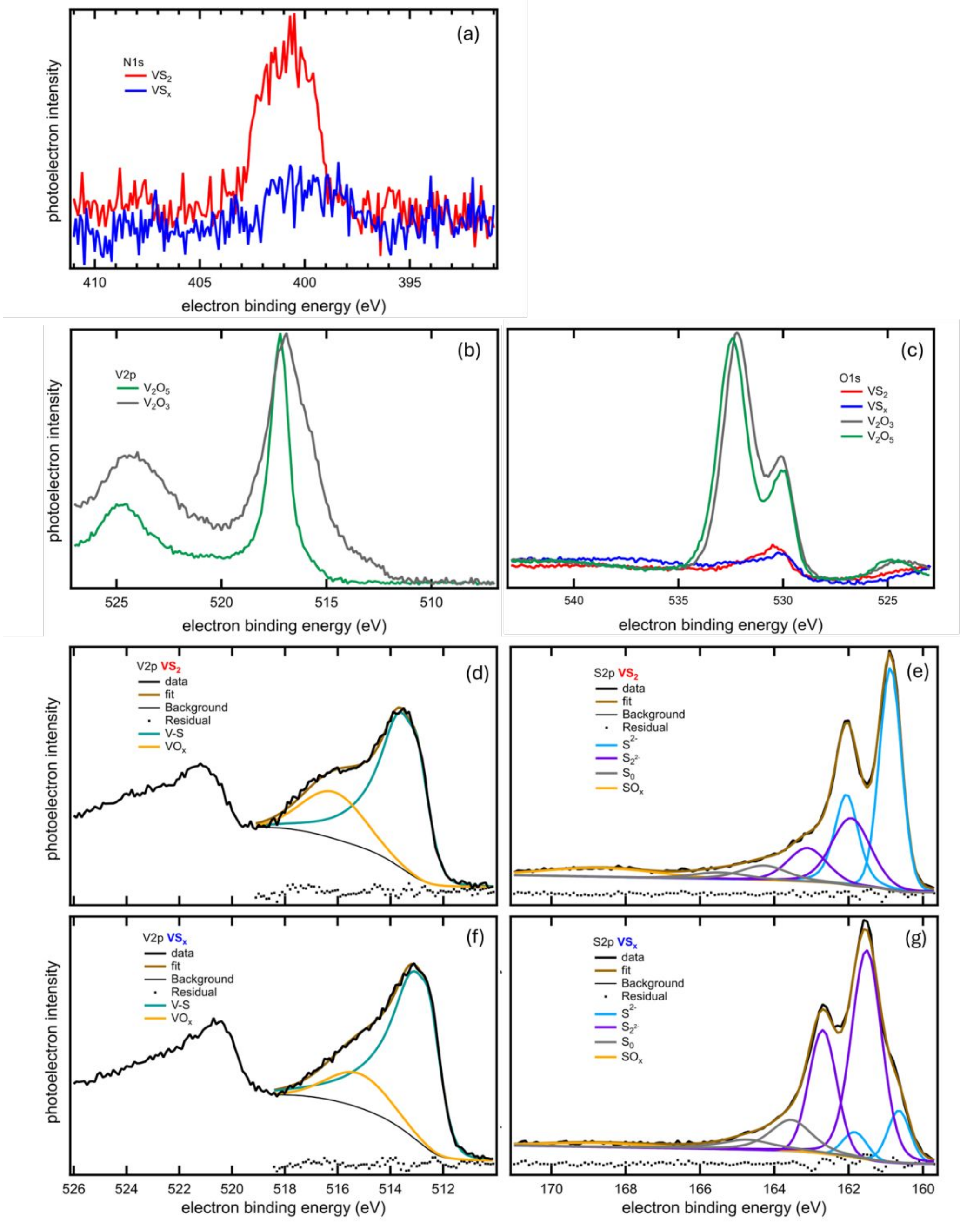

Figure S9. (a) XPS N1s spectra of VS_2 and VS_x, (b) V2p spectra of vanadium oxide references, (c) O1s spectra of VS_2, VS_x, and vanadium oxide references, and (d-g), XPS spectra from Main Text Figure 2(b-e) with extended electron binding energy range.

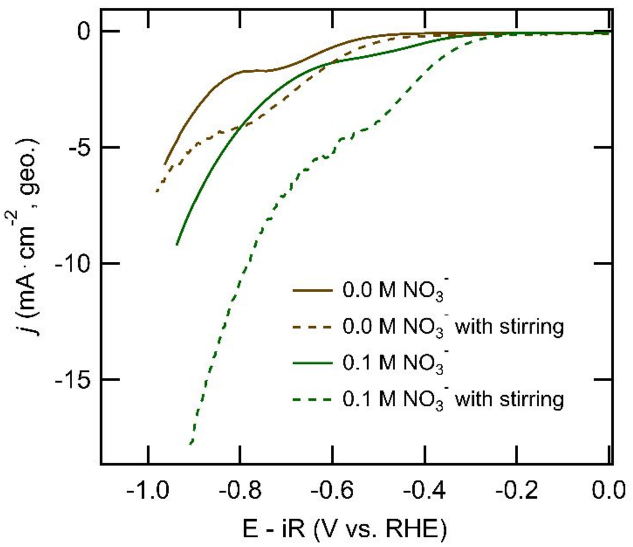

Figure S10. Comparison of VS_x LSVs with stirring (dashed line) and without stirring (solid line). The electrolyte is 0.1 M phosphate buffer (pH 7.0) with or without added 0.1 M KNO_3^-. The stirring rate was 1300 rpm. The scan rate is 20 mV s^{-1}.

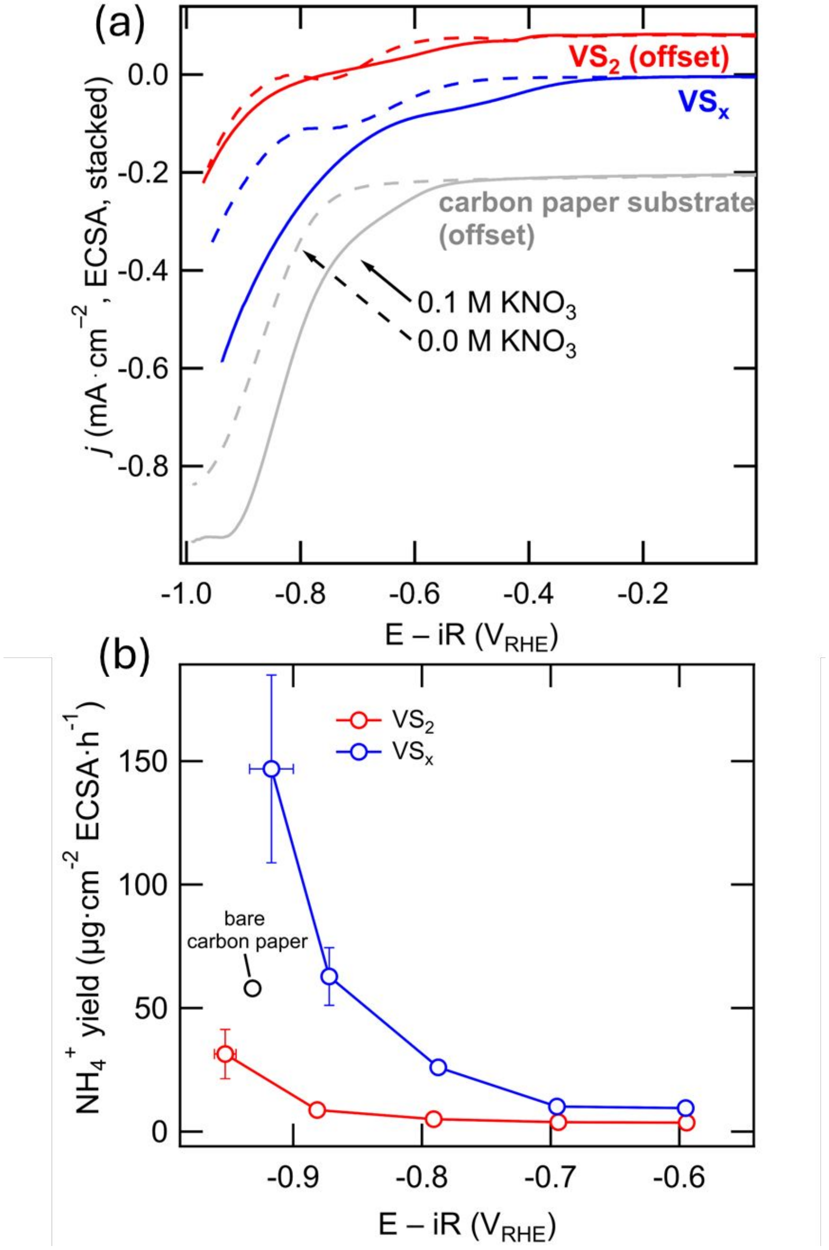

Figure S11. (a) LSV characterization of VS_2, VS_x, and carbon paper substrate data from Main Text Figure 3 with surface area reported as electrochemically active surface area and (b), activity characterization of VS_2, VS_x, and carbon paper substrate data from Main Text Figure 3 with surface area reported as electrochemically active surface area. In (a), The VS_2 trace is offset by $+0.09$ mA·cm^{-2} ECSA, and the carbon paper substrate trace is offset by -0.2 mA·cm^{-2} ECSA.

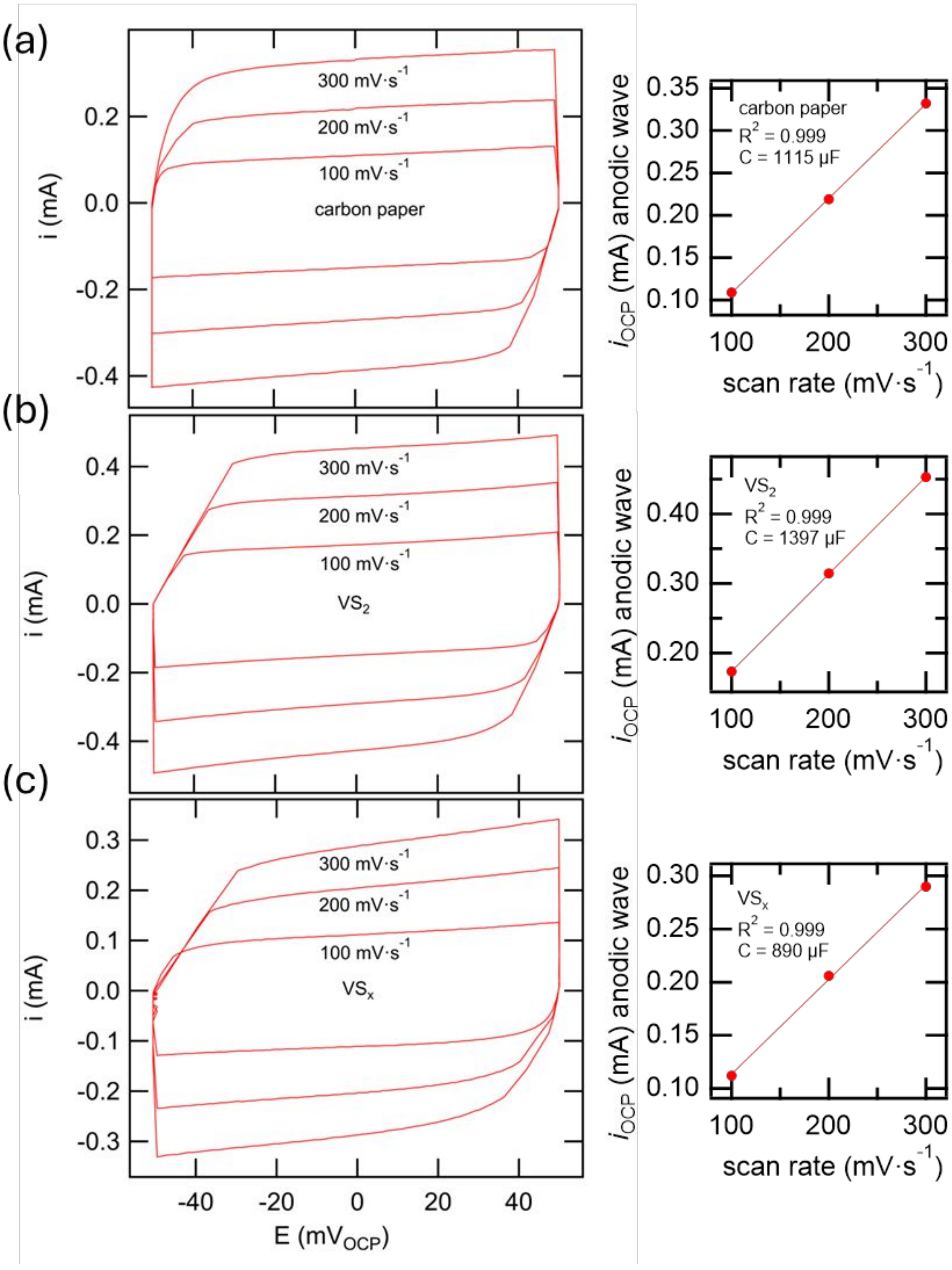

Figure S12. Representative double layer capacitance measurements for determination of electrochemically active surface area (ECSA) of (a) carbon paper, (b) VS_2, and (c) VS_x (0.1 M phosphate buffer (pH 7.0)).

Explanation of ECSA Calculation

The specific capacitance of all electrodes was approximated as 40 $\mu F \cdot cm^{-2}$. The carbon paper substrate, VS_2, and VS_x electrodes had geometric surface areas of 7.33, 2.37 and 1.41 cm^2,

respectively. The measured ECSA of the carbon paper substrate, VS_2, and VS_x electrodes was 27.85, 34.84 and 22.14 cm^2. Thus, the ratio of ECSA to geometric surface area for the carbon paper substrate, VS_2, and VS_x was 3.8, 14.7 and 15.7, respectively.

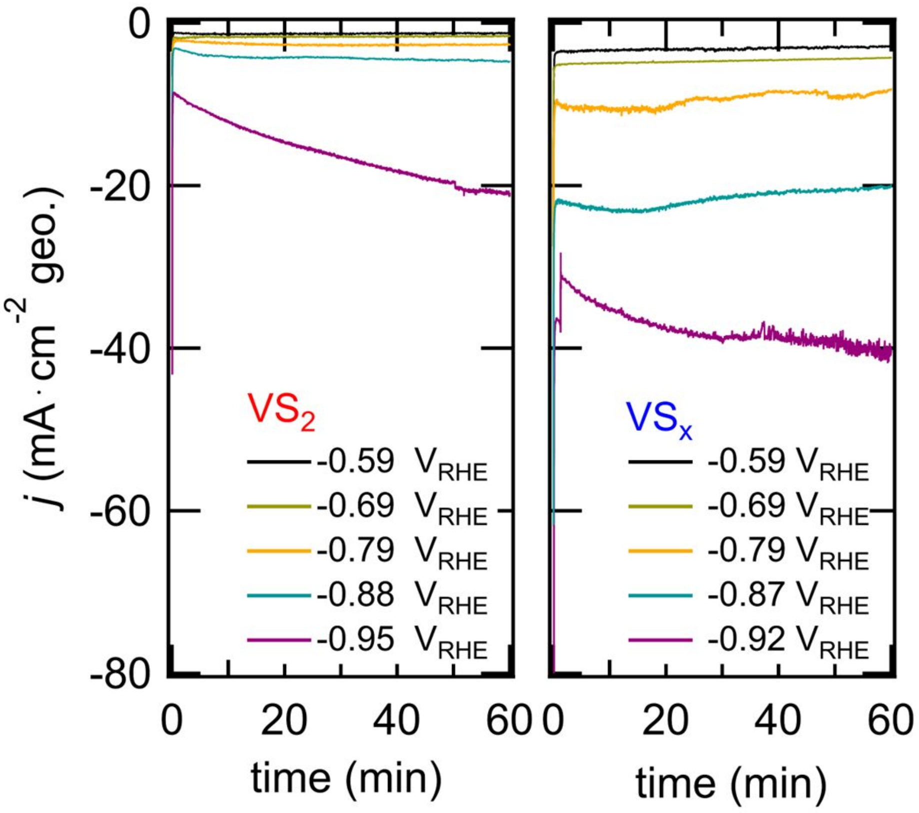

Figure S13. Chronoamperometry traces of VS_2 (left) and VS_x (right) during bulk electrolysis experiments in phosphate-buffered (pH 7.0, 0.1 M) KNO_3 (0.1 M) electrolyte. The experiment was carried out in an H-cell with stirring.

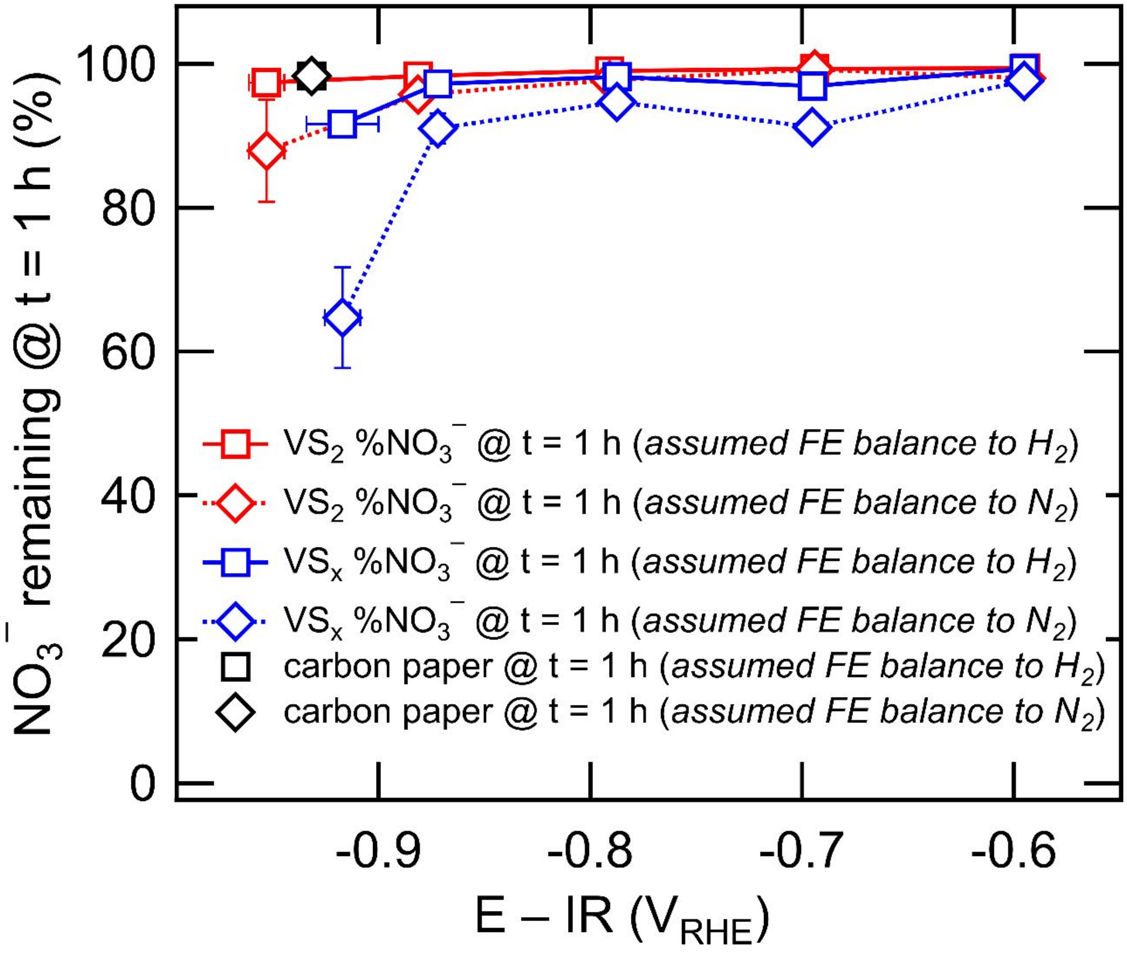

Figure S14. Estimate of NO_3^- remaining upon completion of bulk electrolysis experiments described in the Main Text.

Explanation of Calculation of Remaining NO_3^- Upon Completion of Bulk Electrolysis Experiments

The "assumed FE balance to H_2" estimate uses only the measured concentrations of NH_4^+ and NO_2^- to calculate NO_3^- remaining, with the assumption that the only other electrolysis product is H_2. The "assumed FE balance to N_2" estimate uses both the measured concentrations of NH_4^+ and NO_2^- and the charge passed to estimate the remaining NO_3^-, with the assumption that charge which does not go to formation of NH_4^+ and NO_2^- goes to formation of N_2 by NO_3^- reduction.

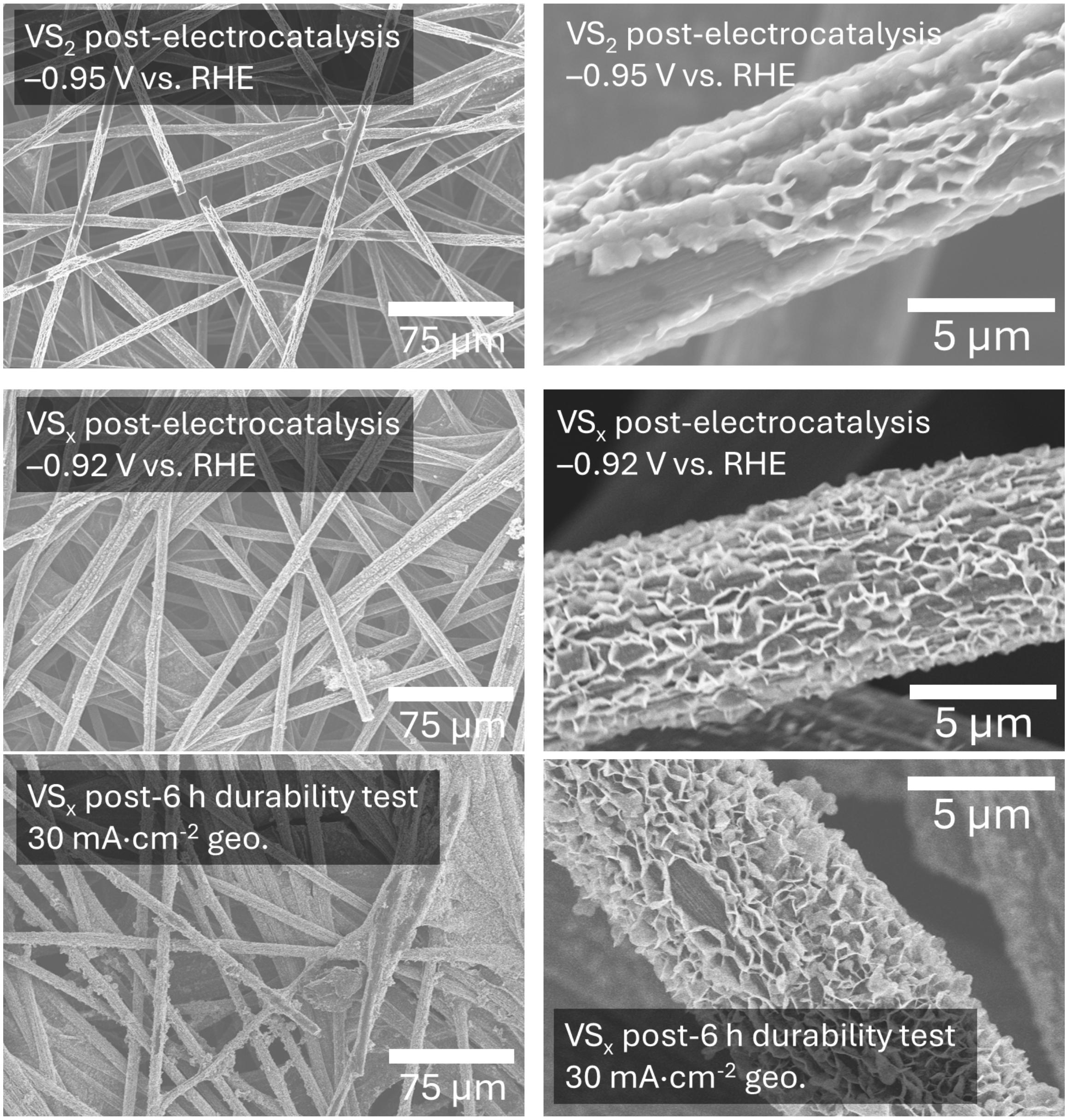

Figure S15. SEM images of post-electrocatalysis VS_2 (top) and VS_x (middle) at the most cathodic potentials tested. The VS_2 shows significant morphology changes, while the VS_x does not show significant morphology changes. Also shown is VS_x after a 6 h, j = 30 mA·cm^{-2}geo. durability test (bottom) which shows some evidence of flaking.

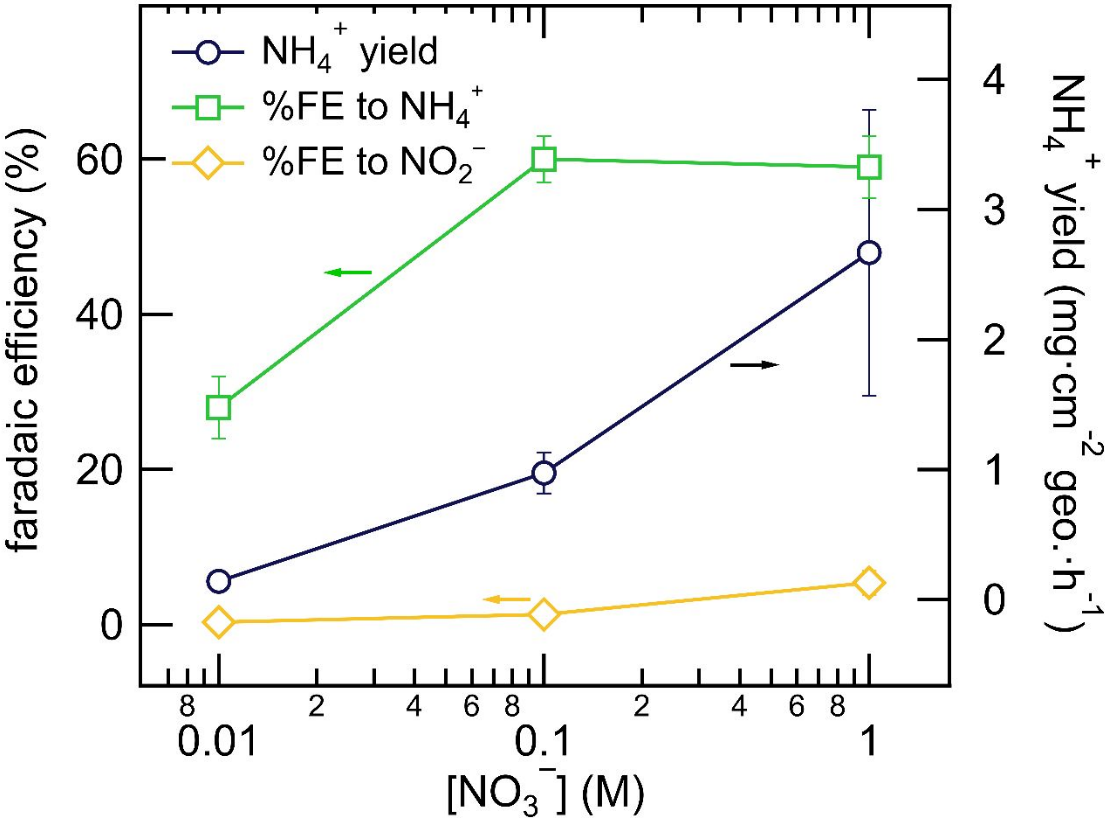

Figure S16. VS_x NO_3RR selectivity and activity concentration dependance at nominal E = 0.9 V_{RHE} with 85% active IR correction. The calculated E for the 0.01, 0.1, and 1.0 M KNO_3^- concentration conditions is 0.88, 0.87, and 0.87 V_{RHE}, respectively. All experiments were performed in 0.1 M phosphate buffer (pH 7.0). The nitrate total percentage remaining in this case for the 0.01, 0.1, and 1.0 M KNO_3^- concentration conditions is 93 ± 1 %, 97 ± 1 %, and 99 ± 1 %, respectively, assuming current which does not result in the generation of NO_2^- and NH_4^+ goes to the HER side reaction.

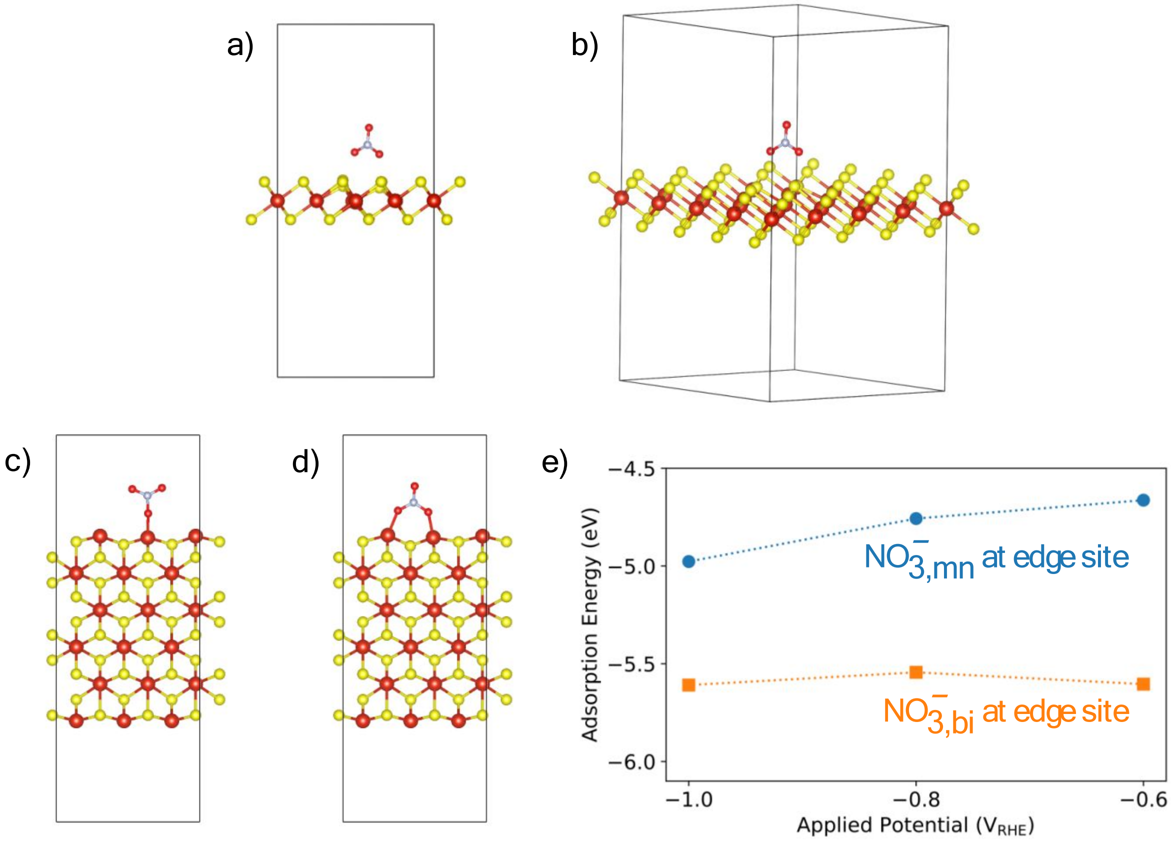

Figure S17. (a-b) Views of models of fully coordinated 1T-phase VS_2 surface structures where NO_3^- does not adsorb (unconverged). (c-d) Edge model of 1T-phase VS_2 with NO_3^- adsorbed in monodentate (c) and bidentate (d) configurations. Structures shown are converged at -0.6 V_{RHE}. (e) Calculated NO_3^- adsorption energies at VS_2 edge, showing overly favorable binding energies.

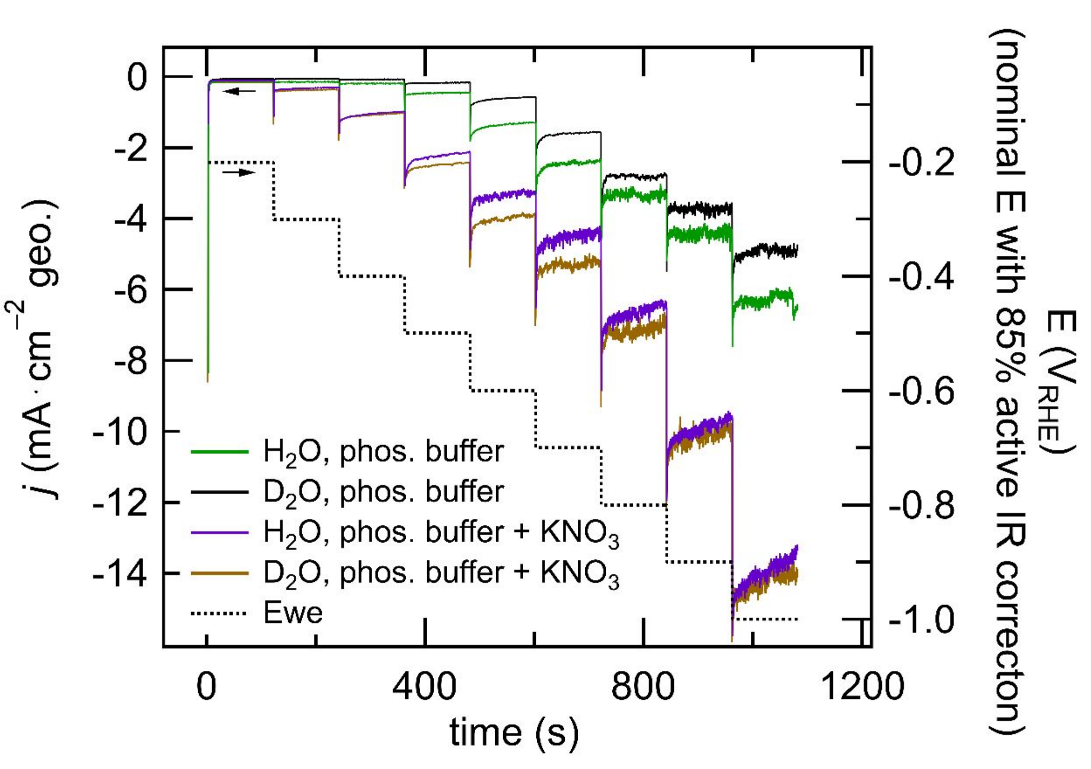

Figure S18. Stepped chronoamperometry of VS_x in H_2O and D_2O-based electrolyte (pH 7.0, 0.1 M phosphate buffer), with or without 0.1 M KNO_3, with forced convection.

Supporting Information References

(1) Mulazzi, M.; Chainani, A.; Katayama, N.; Eguchi, R.; Matsunami, M.; Ohashi, H.; Senba, Y.; Nohara, M.; Uchida, M.; Takagi, H.; et al. Absence of nesting in the charge-density-wave system1T-VS2as seen by photoelectron spectroscopy. *Physical Review B* **2010**, *82*, 075130.
(2) Yu, M.; Huang, H.; Hu, J.; Wang, S.; Li, J.; Wang, D. Vanadium defect-engineering in molybdenum disulfide for electrochemical nitrate reduction. *Journal of Materials Chemistry A* **2022**, *10*, 23990-23997.
(3) Adesope, Q.; Altafi, M. K.; Amagbor, S. C.; Balogun, K.; Guragain, M.; Kafle, A.; Mesilov, V.; D'Souza, F.; Cundari, T. R.; Kelber, J. A. Electrocatalytic Reduction of Nitrate to Ammonia at Oxidized Vanadium Surfaces with V(3+) and V(4+) Oxidation States. *Journal of The Electrochemical Society* **2024**, *171*, 076504.
(4) Mahmood, S.; Bilal, A.; Ammar, M.; Khan, S.; Afshan, N.; Alduhaish, O.; Hassan, N.; Ashraf, G. A.; Bahadur, A.; Fow, K. L.; et al. Highly selective and stable electrochemical reduction of nitrate to ammonia using VO2-x/CuF catalyst with oxygen vacancies. *Journal of Power Sources* **2024**, *608*, 234644.
(5) Wang, G.; Shen, P.; Chen, K.; Guo, Y.; Zhao, X.; Chu, K. Rare-earth La-doped VS2−x for electrochemical nitrate reduction to ammonia. *Inorganic Chemistry Frontiers* **2023**, *10*, 2014-2021.
(6) Feng, J.; Sun, X.; Wu, C.; Peng, L.; Lin, C.; Hu, S.; Yang, J.; Xie, Y. Metallic few-layered VS2 ultrathin nanosheets: high two-dimensional conductivity for in-plane supercapacitors. *J Am Chem Soc* **2011**, *133*, 17832-17838.
(7) Zhang, J.; Zhang, C.; Wang, Z.; Zhu, J.; Wen, Z.; Zhao, X.; Zhang, X.; Xu, J.; Lu, Z. Synergistic Interlayer and Defect Engineering in VS2 Nanosheets toward Efficient Electrocatalytic Hydrogen Evolution Reaction. *Small* **2018**, *14*, 1703098.
